# Is the Prosthetic Homologue Necessary for Embodiment?

**DOI:** 10.3389/fnbot.2016.00021

**Published:** 2016-12-20

**Authors:** Chelsea Dornfeld, Michelle Swanston, Joseph Cassella, Casey Beasley, Jacob Green, Yonatan Moshayev, Michael Wininger

**Affiliations:** ^1^Prosthetics and Orthotics Program, University of HartfordWest Hartford, CT, USA; ^2^ABC Prosthetics and OrthoticsOrlando, FL, USA; ^3^Mountain Orthotic and Prosthetic ServicesLake Placid, NY, USA; ^4^New England Orthotics and Prosthetics SystemsBranford, CT, USA; ^5^Hanger ClinicEast Syracuse, NY, USA; ^6^Hanger ClinicSomersworth, NH, USA; ^7^Orthocraft, Inc.Brooklyn, NY, USA; ^8^Department of Biostatistics, Yale School of Public Health, Yale UniversityNew Haven, CT, USA; ^9^Cooperative Studies Program, Department of Veterans AffairsWest Haven, CT, USA

**Keywords:** prosthetics, embodiment, aesthetics, human-machine interface, design

## Abstract

Embodiment is the process by which patients with limb loss come to accept their peripheral device as a natural extension of self. However, there is little guidance as to how exacting the prosthesis must be in order for embodiment to take place: is it necessary for the prosthetic hand to look just like the absent hand? Here, we describe a protocol for testing whether an individual would select a hand that looks like their own from among a selection of five hands, and whether the hand selection (regardless of homology) is consistent across multiple exposures to the same (but reordered) set of candidate hands. Pilot results using healthy volunteers reveals that hand selection is only modestly consistent, and that selection of the prosthetic homologue is atypical (61 of 192 total exposures). Our protocol can be executed in minutes, and makes use of readily available equipment and softwares. We present both a face-to-face and a virtual protocol, for maximum flexibility of implementation.

## Introduction

For those with limb loss, prosthetic technology is the target intervention for restoring quality of life (Murray, 2008). However, notwithstanding the ever-improving functionality—and despite the high cost (Zuniga et al., 2015) of an investment in a prosthetic limb—, device rejection rates remain formidable: typical reports are between 20 and 30%, and as high as 50% in some patient populations (Postema et al., 1999; Datta et al., 2004; Biddiss E. A. and Chau T. T., 2007; Biddiss E. and Chau T., 2007; Castellini et al., 2014). Cosmetic appeal, in particular, is considered a design factor of critical importance (Roeschlein and Domholdt, 1989; Gaine et al., 1997). But “cosmetics” is a vague notion, with little extant research to guide future prosthesis design. While it is well-known that color-matching to a patient's skin tone leads to greater device satisfaction (Derwentwood, 1917; Brown, 1947; Weinberg and De Hinrichs, 1952; Bryson, 1965; Newell et al., 1974; Pillet, 1983; Pohjolainen et al., 1990; Campbell et al., 1992; Pereira et al., 1996; Leow et al., 2006; Ehrsson et al., 2008; Kini et al., 2010), there is as yet only a few studies describing the importance of form factor on embodiment (Lamb, 2004; Kini et al., 2010; Jaidev et al., 2013; Raghu et al., 2013; Kamble et al., 2014).

Here, we ask a question that has hitherto not been asked: how “self-like” must a prosthetic hand be in order to be acceptable to the user? The context for the question is as follows: prosthetic hands are expensive and disused for reasons related to lack of embodiment, and cosmetic technology has progressed to where a skilled technologist can make an identical replica of absent anatomy (Altman, 2016), but typically at the cost of intensive resource investment. Thus, it is incumbent to ask: how exacting must the prosthesis be? We propose a user-preference survey as a platform for testing the need for a prosthetic homologue.

Our protocol builds on previous studies on prosthetic aesthetics, notably lines of inquiry into the uncanny valley (Gee et al., 2005; Cabibihan et al., 2006; Poliakoff et al., 2013), and user preference in cosmetic appeal (Millstein et al., 1986; Carrozza et al., 2005; Kargov et al., 2007; Dalley et al., 2009), and in particular, we believe we put into an empirical framework one of the most-oft recognized design priorities in prosthetic manufacture (Biddiss E. A. and Chau T. T., 2007; Biddiss E. and Chau T., 2007). The protocol presented here in extends naturally on these studies, in doing so, provides an answer to a critical question: given a set of candidate hands which are *all* equally lifelike, would the user manifest preference for their own hand, and would their preferences be consistent? Whereas these other protocols present hands that of variously life-like or not-so-life-like character—and the degree of life-likeness is a matter of subjectivity—, our protocol presents only images collected from real humans, which are therefore inherently life-like. This protocol best suits those investigators whose objective is to measure the (putative) importance of prosthetic homology to upper limb prosthetic patients. This protocol is designed to test the following hypotheses: (1) whether individuals show consistent preferences for hand designs, and (2) whether individuals show preferences for hand designs that are homologous to their own hand. Here, we present a full protocol and preliminary results from pilot testing in our laboratory.

## Materials and equipment

### Face-to-face

Implementation of this protocol in the face-to-face setting will require the following materials:

1. Computer with requisite softwares. The computer can be of any model; an entry-level laptop or desktop should be adequate to support this experiment. The following softwares are recommended:
  - An image manipulation software: Adobe Photoshop, GNU Image Manipulation Program (GIMP), Corel PaintShop or similar. The purpose of this software is to create a binarized image of the hand based on the photo, to standardize the image size, and to count the proportion of black pixels (“pixel count”).
  - A spreadsheet software: Microsoft Excel, OpenOffice Calc, GoogleDocs Spreadsheet, or similar. The purpose of this software is to create a convenient reference list for sorting images by pixel count.
  - A document or presentation design software: Microsoft PowerPoint, Microsoft Word, or similar. The purpose of this software is to create a series of hand line-ups for the subject to review.

We note that for experienced programmers, this suite of softwares can be obviated by use of a numerical computing environment: Matlab, Octave, R or similar.

2. Camera (optional: Tripod). This camera will be used to take a picture of the hand resting on the backdrop.
3. Backdrop for photographing hand. This can be cloth swatch of fabric with high-contrast color to the subject's skin tone. However, because skin tone can vary between subjects, a light box may provide the most reliable and effective backdrop.
4. Image bank with pre-binarized hand silhouettes. Subjects will be asked to review a number of hand images; ideally, these hands should be somewhat comparable to the subject's own hand. Therefore, it is vital to have a number of hand samples prepared in advance of the first subject. We recommend a minimum of 10 hands. As new subjects participate in the study, their images can be added to the bank. All hands should have similar postures, so as to eliminate this source of variance.
5. Data collection forms. The purpose of the data collection are to capture both the aesthetic preferences of the subject, and also potentially relevant explanatory variables. Two types of surveys are suggested:
  - Hand preference data: Most likely a single-page is sufficient; the purpose of this survey is to document which hands were selected by the patient.
  - Demographic and psychological profile: Key variables to collect include age, sex, hand dominance, and history of hand, or arm pathologies. We recommend that some measure of anxiety or body image be used. Particularly well-regarded instruments include the Brief Fear of Negative Evaluation Scale (Leary, 1983), and the Appearance Schemas Inventory: Personal Opinions Questionnaire (Cash and Labarge, 1996). It may also be desirable to ask direct questions about the importance of aesthetics, or design priorities in the hypothetical scenario where the subject needs a prosthetic hand.
6. Large screen with arm holes. The screen can be made of any material; a standard 36″ × 48″ tri-fold poster board is adequate. The screen should be large enough to prevent the subject from seeing the workspace around their hands.
7. Decoys (optional). A small number of physical objects for manipulation may be used to distract the subject in case the procedure takes more than a few minutes to implement. In place of physical objects, the subjects could be presented with a sham survey or puzzle to solve.

### Virtual

Implementation of this protocol in the virtual setting will require the following materials:

1. Two internet-connected devices with video. Both the investigator and the subject must have hardware to support exchange via teleconference.
  - Investigator: Most likely this be a laptop or desktop. Softwares will be needed as described above (Face-to-Face, Item 1).
  - Subject: This can be either a computer or a handheld device, e.g., smartphone or tablet. Most likely, no additional softwares will be needed. It is important to reduce the software requirements on the subject so as to make the study accessible to a wide participant pool.

We note that both parties will need access to a mutually compatible teleconferencing software, e.g., Skype, Webex, GoToMeeting or similar.

2. Backdrop for photographing hand. Same as above. This can be accomplished via a drapery, wallpaper or paint in solid color, or other plain, flat item.
3. Image bank. Same as above (Face-to-Face, Item 4).
4. Data collection forms. Same as above (Face-to-Face, Item 5). These can be administered in a way that suits the investigators and the study, i.e., it is conceivable that these data can be collected verbally and noted by the investigators, or that these data can be collected electronically, e.g., via email, text, or web-based survey.
5. Decoys (optional). Same as above (Face-to-Face, Item 7), with the logistical constraint that physical objects cannot be transferred to a remotely participating subject.

## Stepwise procedures

### Face-to-face

**Figure 1:**
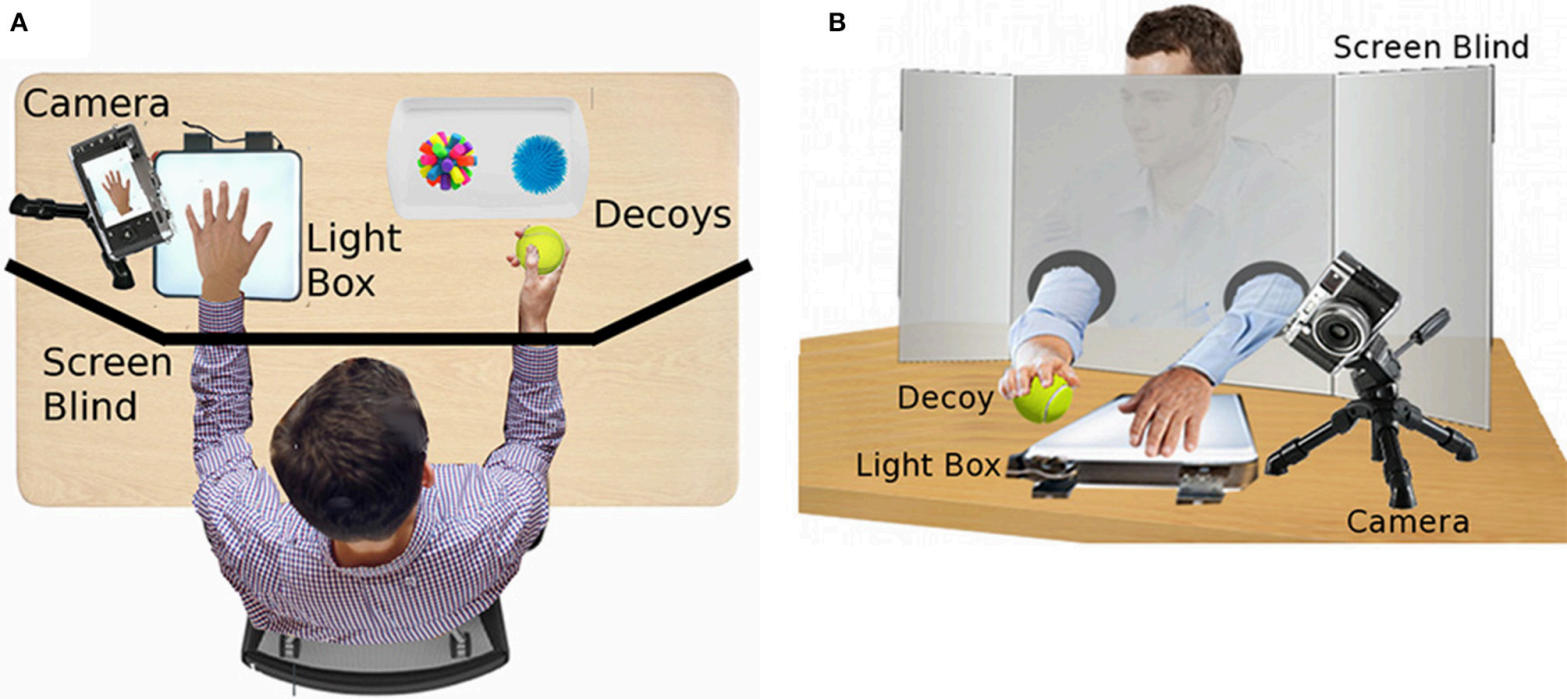
**Diagram of Face-to-Face setup:** Top view **(A)** and Front view **(B)**.

1. Prior to potential subject arrival, set up all materials as shown in Figure 1.
  - The subject should see only a chair and the screen.
  - Behind the screen should be the light box, the decoys, and the camera.
2. Initiate the Informed Consent process. Advise the potential subject that some of the activities of the study will include blinding; all details will be revealed at the conclusion of the session.
3. Seat subject at the table, and place their arms through the holes in the screen with palms down on the table.
  - Ask them which hand is their dominant hand.
  - Place the decoys near to their non-dominant hand and ask them to feel around until they can find the decoys.
  - Investigator should spread the fingers to conform to the template of the hands in the hand bank.

^*^Steps ii and iii should be performed simultaneously: Step ii is intended to distract the subject from Step iii.

**Figure 2:**
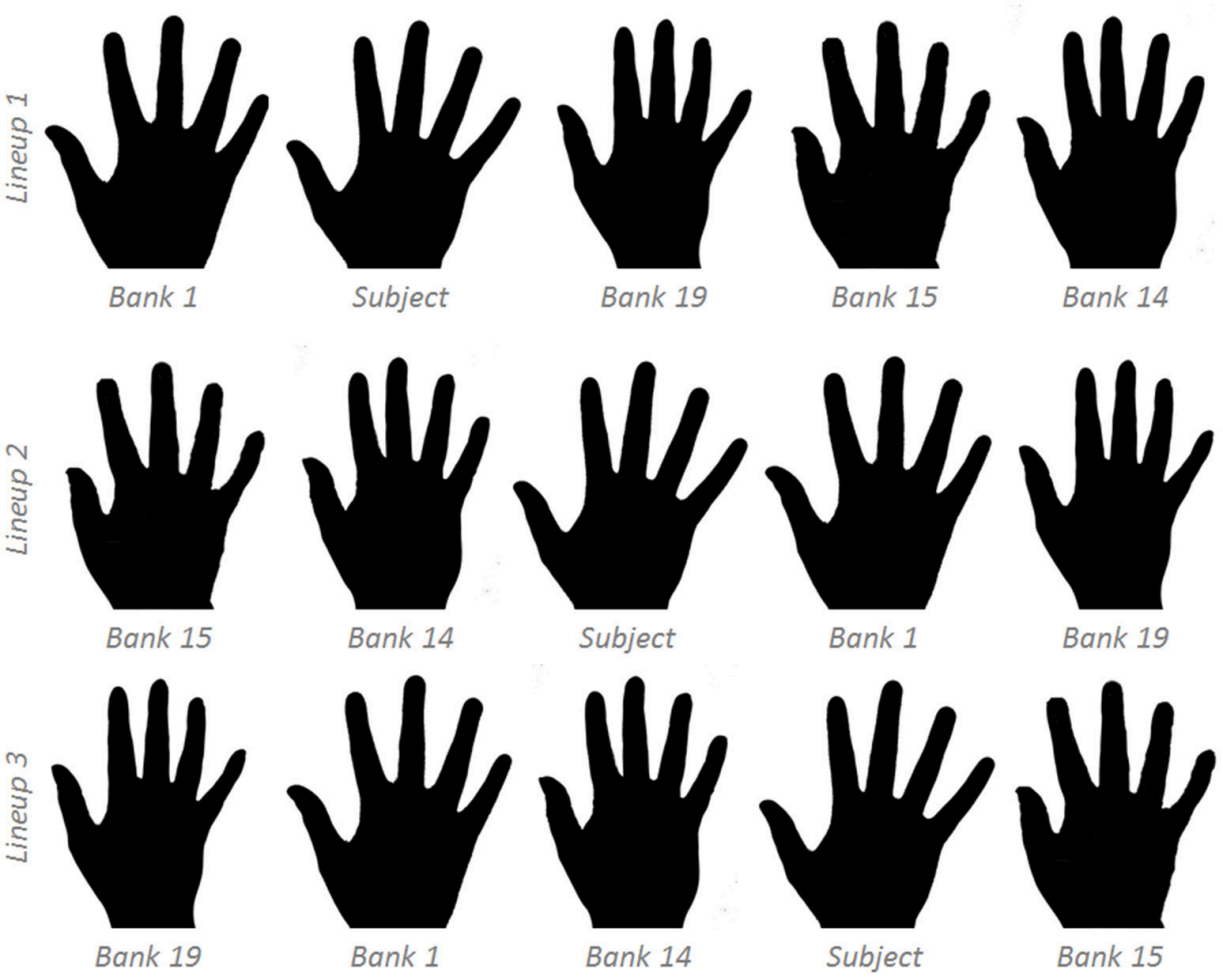
**Sample hand line-ups:** four hands from the image bank were matched to the subject's hand. These lineups will be repeated for six total lineups.

4. Engage the decoys with the non-dominant hand. Take the photo of the dominant hand; camera should capture the dorsal aspect.
5. Binarize the photo to standard specifications (see Supplementary Material).
6. Select four hands from the image bank with greatest similarity to the subject's hand.
  - Select by pixel count.
  - Select two hands with greater pixel count and two with lesser pixel count.
7. Organize the five images in a row in random order, repeat for three total lineups. Copy these lineups once, occupying six total slides (Figure 2). Take note of which image is placed where. Each lineup contains the same five images, just in three different orders (Slide 1 and 4 have the same exact ordering; Slides 2 and 5, and Slides 3 and 6, as well).
8. Present the slides to the subject, and instruct them: “We will show you six slides; each slide has one lineup of five hand images. Please identify the hand that you find most aesthetically pleasing in each lineup. In particular, we want to know: if you had to receive a prosthetic hand, which hand would you most like your prosthesis to look like?” Note all six choices.
9. Solicit the participant to complete the demographic and psychologic surveys.
10. Thank the subject for their completion of the Protocol and explain the study for them. It is permissible to review their results.

### Virtual

**Figure 3:**
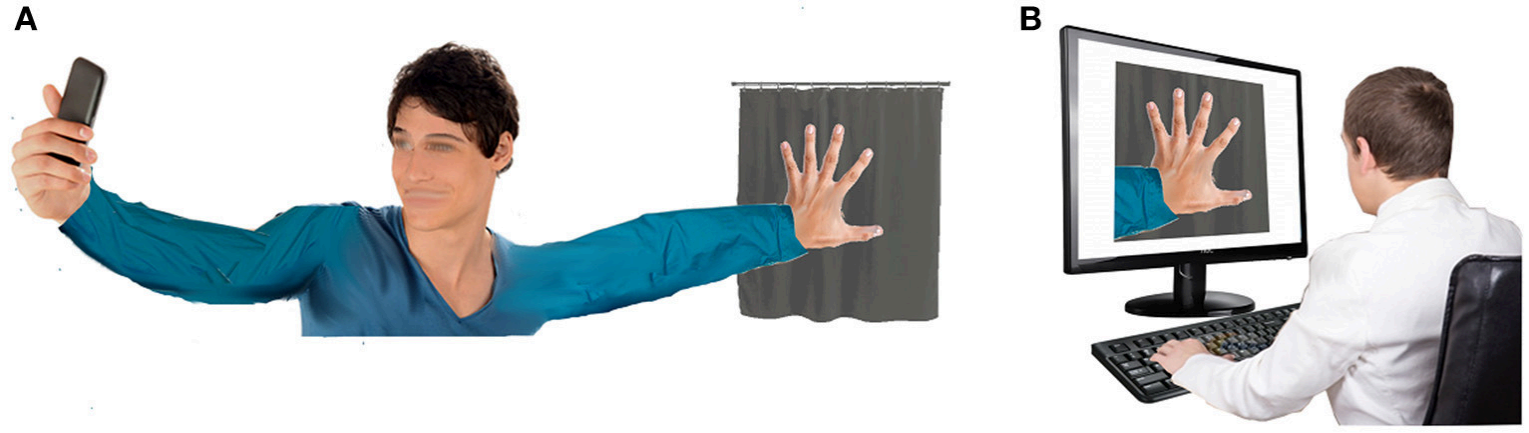
**Diagram of Virtual setup:** Subject view **(A)** and Investigator view **(B)**.

1. Upon logging on, initiate the Informed Consent process. Same as above (Face-to-Face, Step 2).
2. Ask subject to pose their hand and take a photo (as in Figure 3). Coach the subject through the process until the hand meets target posture. Subject will need to transmit photo to the study team.
3. Binarize the photo (Face-to-Face, Step 5).
4. Create the slide deck and present to subject (Face-to-Face, Steps 6–8).
5. Complete demographic and psychologic surveys (Face-to-Face, Step 9).
6. Session close (Face-to-Face, Step 10).

## Anticipated results

### Image bank

We have recruited 20 volunteers for our development of our image bank. We exhibit representative samples of these banked images in Figure 2. Following our Supplement, each image required ~90 s to convert from raw image to binarized image with noted pixel count.

### Preliminary results

**Figure 4:**
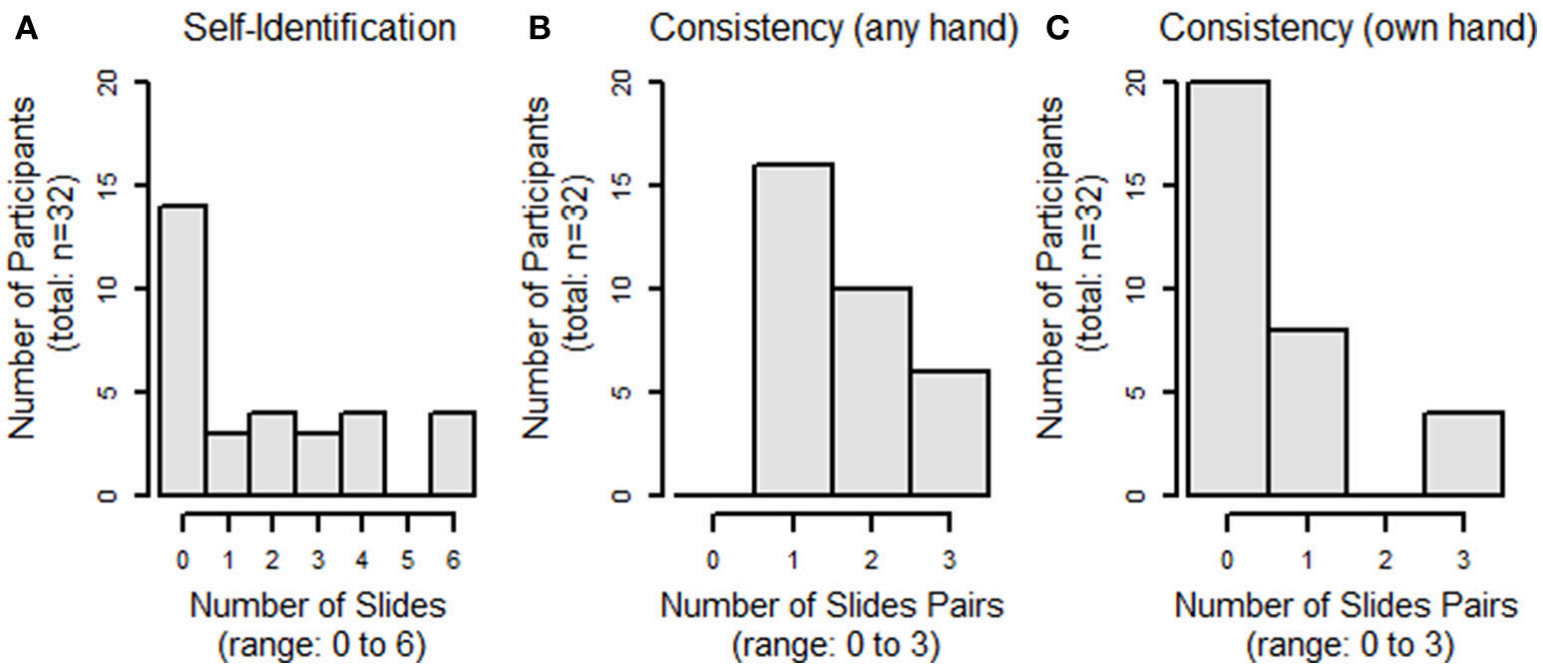
**Histograms of subject selection of their own hand (A)**, consistency within slide pairs **(B)** and rates of one's own hand appearing in matched slide-pairs **(C)**.

We have pilot-tested our Face-to-Face protocol on 32 subjects. Our population comprised healthy volunteers with completely intact anatomy: 13 M/19 F, 26 ± 11 years (range: 19–53 years), all right-hand dominant (with one ambidextrous subject), with an average score on the Brief Fear of Negative Evaluation Score (BFNES) of 33 ± 6 (range: 21–39; range: 0, not at all fearful–60, maximally fearful). Homology scores, i.e., the number of times the subject selected their own hand as the most appealing (max = 6) are shown in Figure 4; the mode value was zero selections of one's own hand (Figure 4A). Consistency scores, i.e., the number of times the hands selected in lineup pairs were the same (max = 3) were as follows: 1 (*n* = 16), 2 (*n* = 10), and 3 (*n* = 6; Figure 4B); all subjects had at least on consistent matching. We note that among those slide-pairs showing consistency within the pair, four subjects (12.5%) selected their own hand in all three pairs, while 20 subjects with consistent pairings consistently picked a hand that was not their own (Figure 4C).

**Figure 5:**
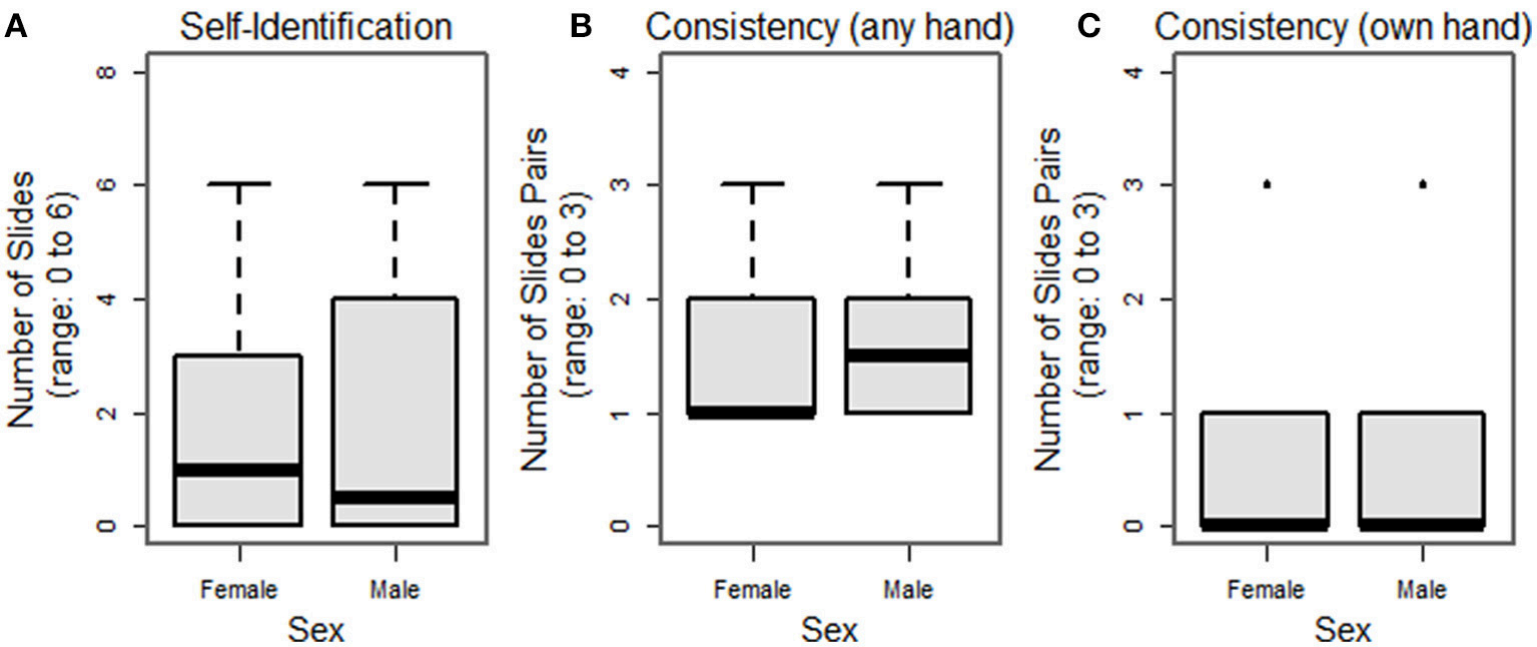
**Breakout of selection of one's own hand (A)**, consistency within slide pairs **(B)** and rates of one's own hand appearing in matched slide pairs **(C)**, breakout by sex.

**Figure 6:**
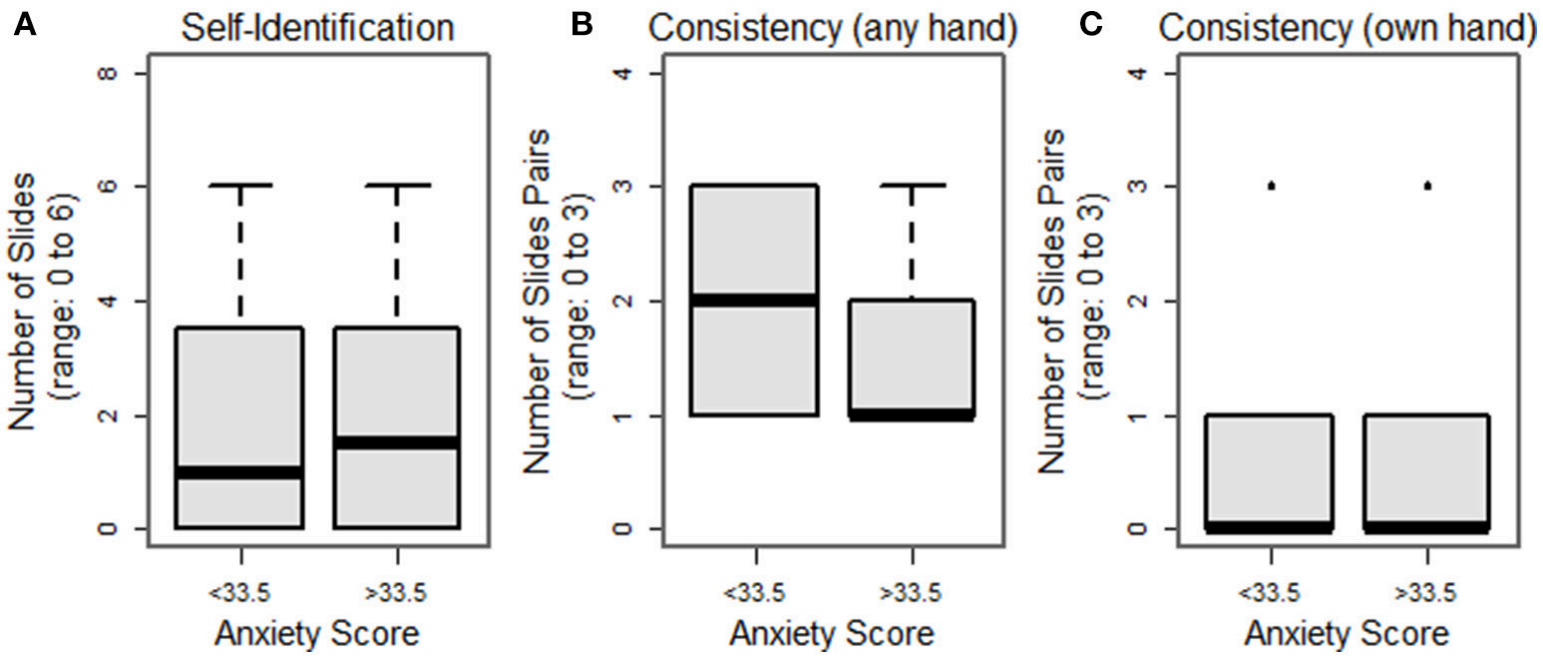
**Breakout of selection of one's own hand (A)**, consistency within slide pairs **(B)** and rates of one's own hand appearing in matched slide pairs **(C)**, breakout by Brief Fear of Negative Evaluation Score (BFNES), dichotomized at median observed value.

Preliminary hypothesis testing was performed to assess selection of one's own hand, consistency, and the rate of consistent selection of one's own hand, both according to sex, and score on the BFNES, dichotomized about the median. Due to the non-normality of the data, non-parametric tests were used. None of these analyses revealed significant differences between groups (Figures 5, 6). Given that the frequency selection of one's own hand from the hand bank is low, and does not suggest systematic trends by the variables collected in our pilot study, we draw the inference that the prosthetic homologue may not be a particularly important target in custom prosthesis design for all users. However, there will likely be a sub-group of patients for whom a prosthetic homologue is important, though it is not evident that these patients are readily identifiable by sex or BFNES; our pilot data set is insufficiently powered to test for interaction effects.

## Notes

**Figure 7:**
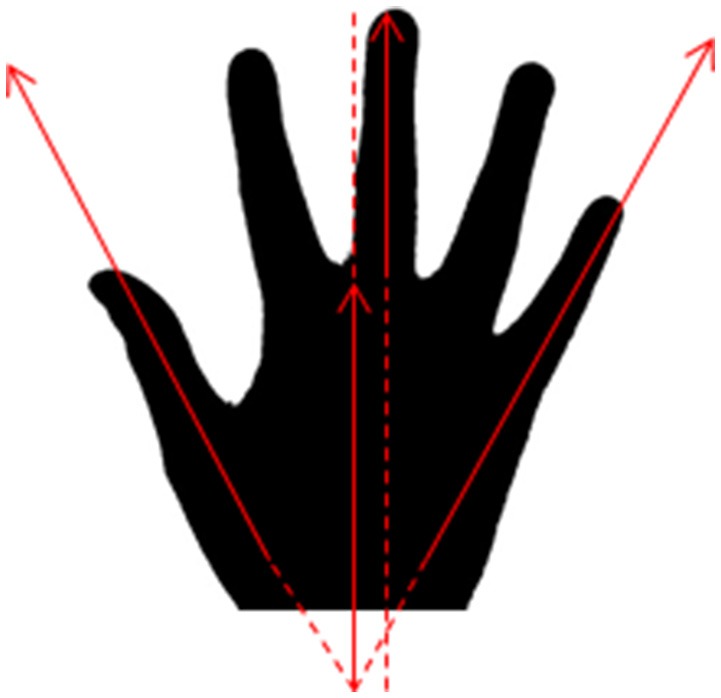
**Suggested hand posture:** thumb and fourth finger spread at 70°–90°, with long finger bisecting. Silhouette truncated approximately at wrist.

We make some notes of relevance to the protocol procedures:

1. It is very important that all hand silhouettes used in this study have the same approximate posture; we recommend fingers spread comfortably: sub-maximally, but so that each finger can be seen for its true shape. In our own work, we target angular separation between thumb and little finger of 70°–90°, with middle finger bisecting and parallel to the radius and ulna (Figure 7).
2. The decoys are an intentional distraction; the nature of the decoy engagement is arbitrary. We provide our subjects with three balls and ask them to sort them in order of stiffness. If they accomplish this task quickly, we ask them to repeat.
3. The primary objective of this study is to test whether subjects will pick hands that look like their own. Therefore, morphology is the variable of highest interest, not size. For this reason, it is critically important to reduce artifact due to size. Images should have uniform canvas size and pixel density within the image processing software (e.g., 2″ × 2″ at 100 dpi), so that the pixel counts will be on the same scale across all images. It is equally important that the hands have similar postures and be windowed to the same landmarks, e.g., the bottom of the image should always (or never) include the wrist bones. We note further that image indexing by pixel attributes is a well-established practice (Gong et al., 1994, 1996; Stehling et al., 2002; Semmlow and Griffel, 2014), but for images comprising binary pixels, the impact on vision and attention (and therefore image selection) could be profound (Papathomas and Julesz, 1989; Gegenfurtner and Hawken, 1996; Papathomas, 2007). Thus, we re-emphasize the importance of scaling images appropriately so that it is the *shape* of the hand, and not the imprint or *size* of the hand that drives the subject's selection.
4. Continuing on Note #3, we designated pixel count as the single parameter best poised to match the participant's hand to the hand bank, based on two justifications: physiological, and practical. As described above, the amount of black versus white content in a binarized image will have substantial impact on the viewer's attention; given this, we wanted to avoid biasing participants to picking hands with extremely high pixel counts: rather, we concluded that by selecting images with similar pixel counts, we could eliminate this source of variance, increasing the likelihood of detecting preferences associated with hand shape. Regarding pragmatics, we could have extracted features from the images, e.g., based on hand contour or finger spread, etc., but discarded these designs on the basis that their complexity would inhibit adoption of this study by a wide range of researchers; by selecting pixel count—a feature that is easily extracted from any image without sophisticated image processing steps—we hope to make this protocol more accessible among the diverse community of investigators with interest in aesthetics and prosthetic design.
5. Hand bank samples were not gender-matched to the participant for the reason that such a constraint might interfere with pixel-count matching. We do not necessarily suggest that gender-matching is undesirable; on the contrary, with an adequately large hand bank, gender matching may be feasible without compromising the pixel-matching. Future work may provide opportunity to explore this alternate design.
6. The tradeoff between Face-to-Face and Virtual experiments is as follows: the Virtual experiment is easier to implement and allows for much easier recruitment and data collection. However, it is less likely that the subjects will remain fully naive to the study objectives: they may make the connection that their hand was being posed for use in the hand lineup, and they may recognize their hand within the lineup. Whether the Virtual experiment truly compromises the subject naïveté remains to be tested, but must be recognized as a possible source of bias. Creative use of decoys will most likely be helpful here.
7. This hand bank is prepared prior to opening the study to enrollment so that there are an ample set of candidate hands to present to first participant. We propose soliciting volunteers to allow their hands to be photographed, and to binarize these images, saving them in a folder for accessing during the data collection phase. We suggest that this pre-collection phase of hand bank creation will give the study critical opportunity to refine their image binarization technique. Over time, our team was able to reduce the time required to binarize an image from 5 min to <30 s; this is a valuable time savings in terms of maintaining participant focus. The reduction in time burden is attributable primarily to improved acquaintance with the routine for binarization; familiarity greatly improves efficiency. We refer the reader to our Supplementalary Materials for step-by-step procedures for image binarization, and recommend MS PowerPoint or equivalent for creating the lineups.

Regarding the implications of this protocol: we firstly recognize the limitations of using a 2-dimensional test (selection of images) for an inherently 3-D problem, i.e., embodiment of a prosthetic device. However, we believe that is a useful first line of inquiry into the necessity and sufficiency of a design criterion based on homology between the prosthetic device and the anatomy that it replaces, and that this is an attractive paradigm than the alternative: 3-D printing a hand for each subject.

It is prudent to discuss two terminologies used here: embodiment, and the homologue. *Embodiment* is generally defined as the integration of an artificial limb into one's own body schema, i.e., the fusion of body and perception (Mulvey et al., 2009). This concept is often invoked in the context of someone asked whether they are able to “make it feel like me.” While our protocol does not utilize actual prostheses, we believe that our prompt to the participants (“Which hand would you most like your prosthesis to look like”) brings our study comfortably into the realm of embodiment. Further, in this work, we are coining the term *prosthetic homologue*, which has heretofore not been described in any scientific literature known to the authors or their collaborators. Our use of the term is meant to pair the concept of similarity (to one's own anatomy; the “homologue”) to anatomical replacement (the prosthesis). We note the concept of homology is occasionally referred to in the literature related to the uncanny valley (Chaminade, 2006; Tondu, 2012; Kaerlein, 2015), but is used in a way that connotes similarity to human anthropomorphism, and not *per se* any one person's anatomy (or one's own anatomy). In many settings, the prosthetic homologue cannot directly be assessed: a limb-deficient individual has no anatomy from which to draw the comparison; the homologue in this case would be an abstract construct, that can only be approached conceptually, but not tested. However, in healthy persons, while there is no direct opportunity for prosthetic replacement, the anatomy is present, can be measured, and preferences in the hypothetical can be tested.

By presenting pilot data as a preliminary result, we intend to provide investigators with basis for formulating study designs, i.e., requisite sample sizes for pre-specified parameters related to statistical power. This entire protocol can be executed via freewares, i.e., at no expense beyond the base materials; given the ubiquity of digital cameras and web access, we believe that this is accessible protocol that can be readily implemented in a wide variety of settings. Lastly, we note that all data collection activities described in this manuscript were performed under the authorization of the University of Hartford Institutional Review Board, following provision of written informed consent.

## Ethics statement

University of Hartford Human Subjects Committee (FWA00003578). All participants signed an informed consent prior to participation in this study.

## Author contributions

MW: Lead investigator, primary study architect. All authors participated in the design of the study, drafting and approval of the manuscript, and take responsibility for its contents.

### Conflict of interest statement

The authors declare that the research was conducted in the absence of any commercial or financial relationships that could be construed as a potential conflict of interest. The reviewer EC and handling Editor declared their shared affiliation, and the handling Editor states that the process nevertheless met the standards of a fair and objective review.
