# Supplementary material for: Is the Prosthetic Homologue Necessary for Embodiment?
Source: Front Neurorobot. 2016 Dec 20;10:21. doi: 10.3389/fnbot.2016.00021 (PMC5168425; doi:10.3389/fnbot.2016.00021)
Supplement: Supplementary file 1 [file Presentation1.PDF]

Supplementary Material

Is the prosthetic homologue necessary or sufficient for embodiment?

Chelsea Dornfeld[1,2], Michelle Swanston[1,3], Joseph Cassella[1,4], Michael Wininger[1,5,6]

*** Correspondence:**
Michael Wininger
Prosthetics & Orthotics Program
University of Hartford
200 Bloomfield Avenue
West Hartford, CT, 06117, USA
wininger@hartford.edu

Supplementary Figures

Supplementary figures follow on subsequent pages.

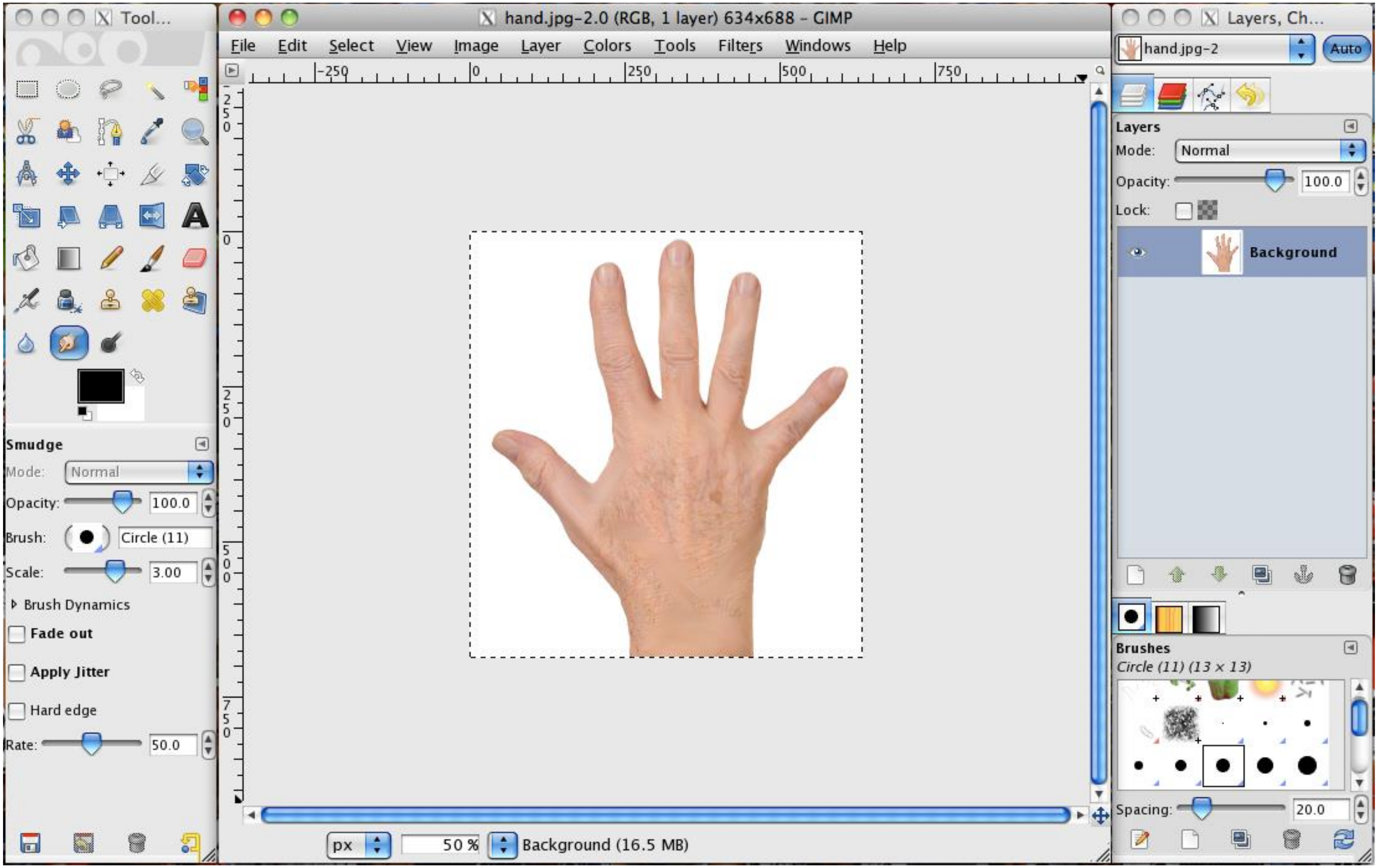

Supplementary Figure 1. STEP 1: Open image in GIMP.

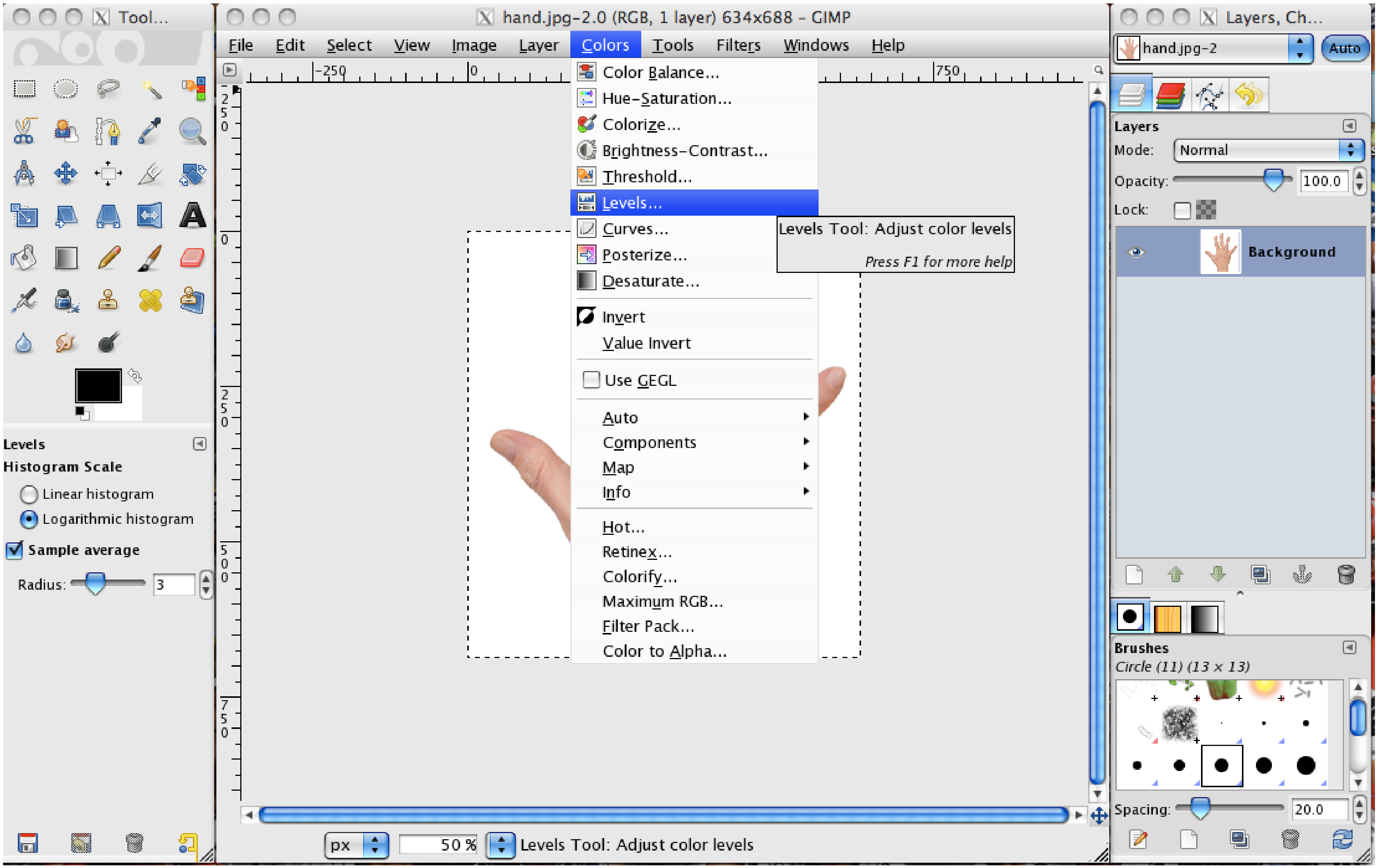

Supplementary Figure 2. STEP 2a: Access the image color level tool from Color > Levels menu.

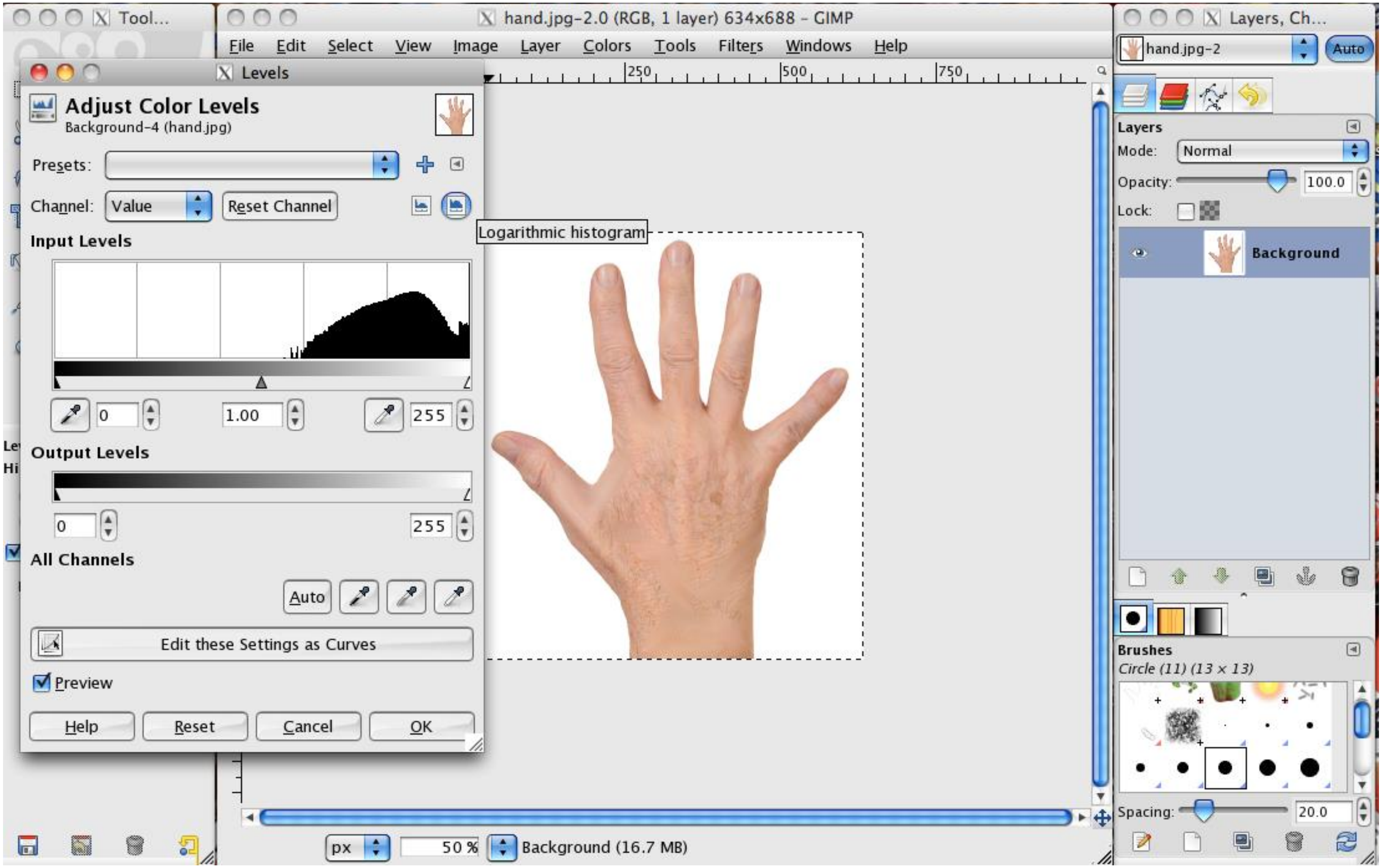

Supplementary Figure 3. STEP 2b: It may be helpful to view the histogram on Log scale (button near top-right).

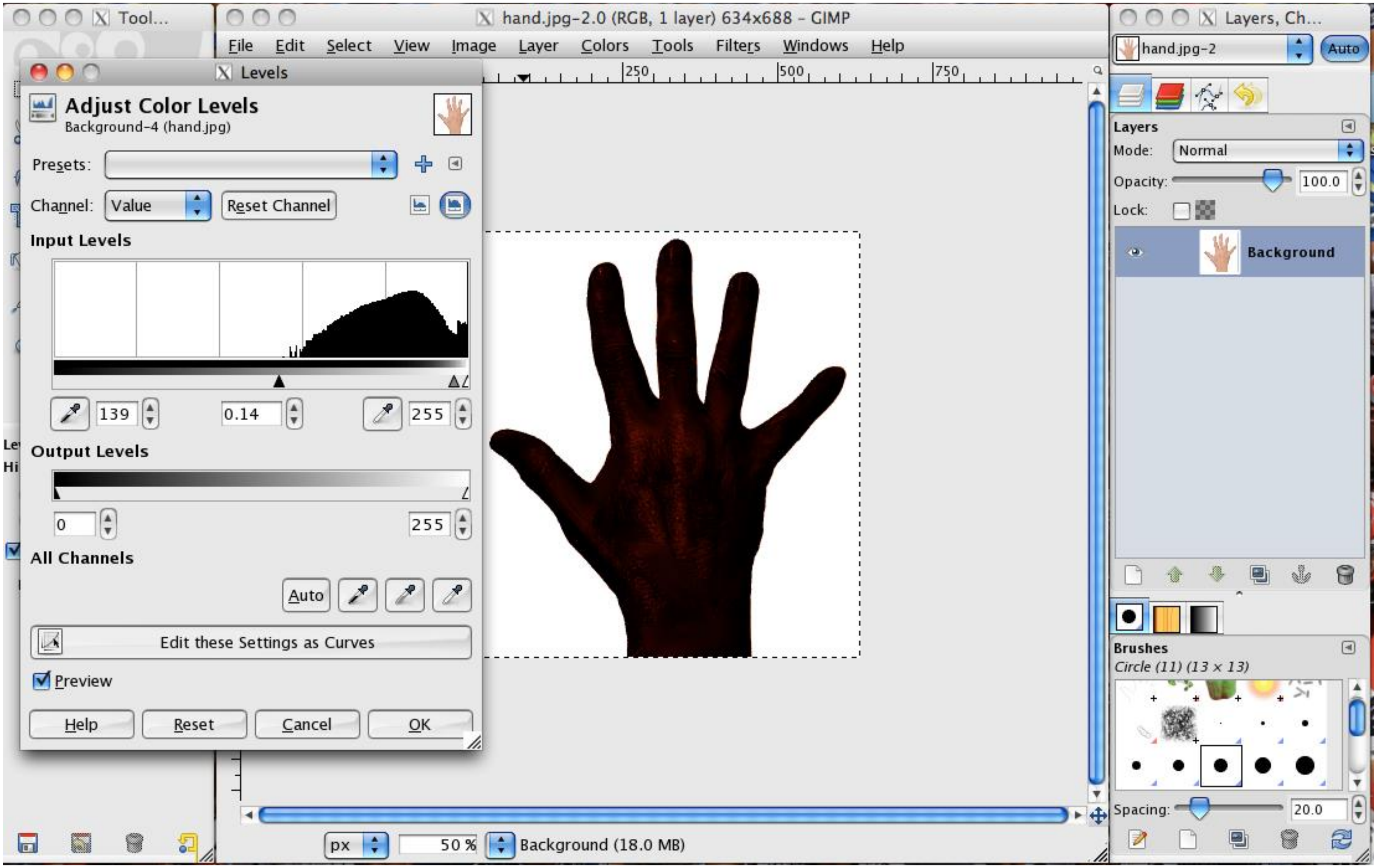

Supplementary Figure 4. STEP 2c: Move the histogram sliders so that the 2nd slider (grey) is in the notch between histogram nodes. If no notch, slide to the far right.

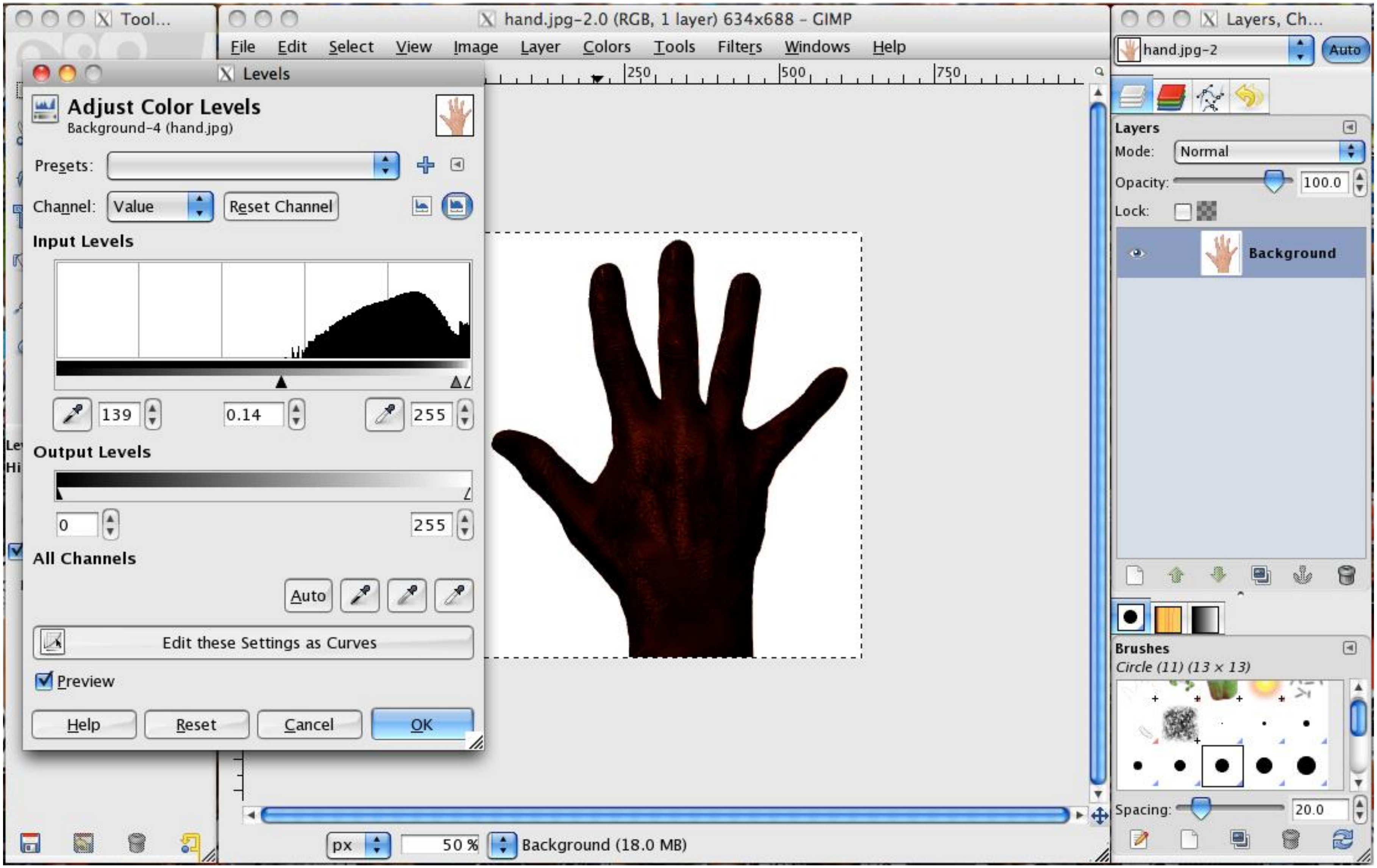

Supplementary Figure 5. STEP 2d: Click 'OK'.

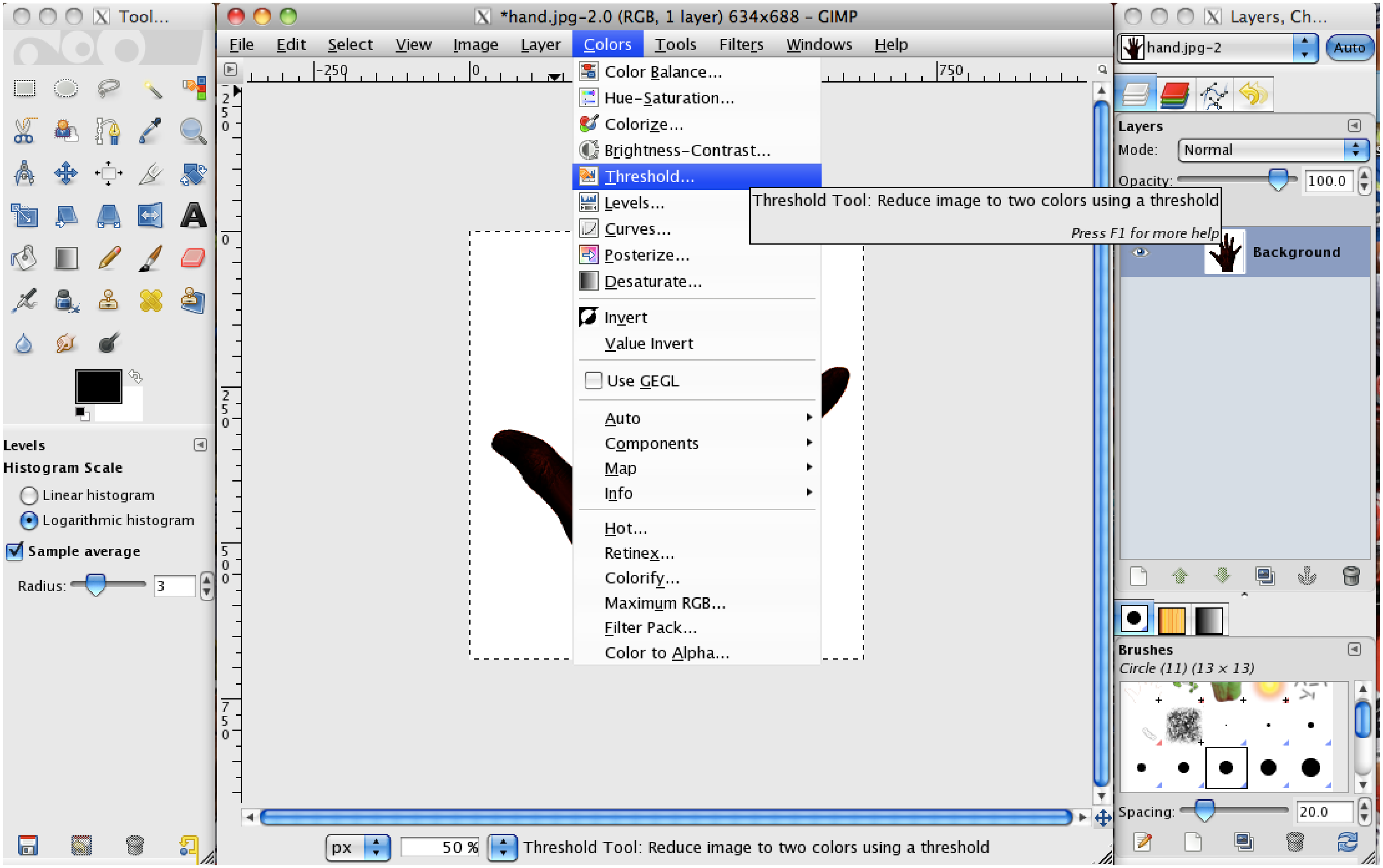

Supplementary Figure 6. STEP 3a: Access the image color threshold tool from Color > Threshold menu.

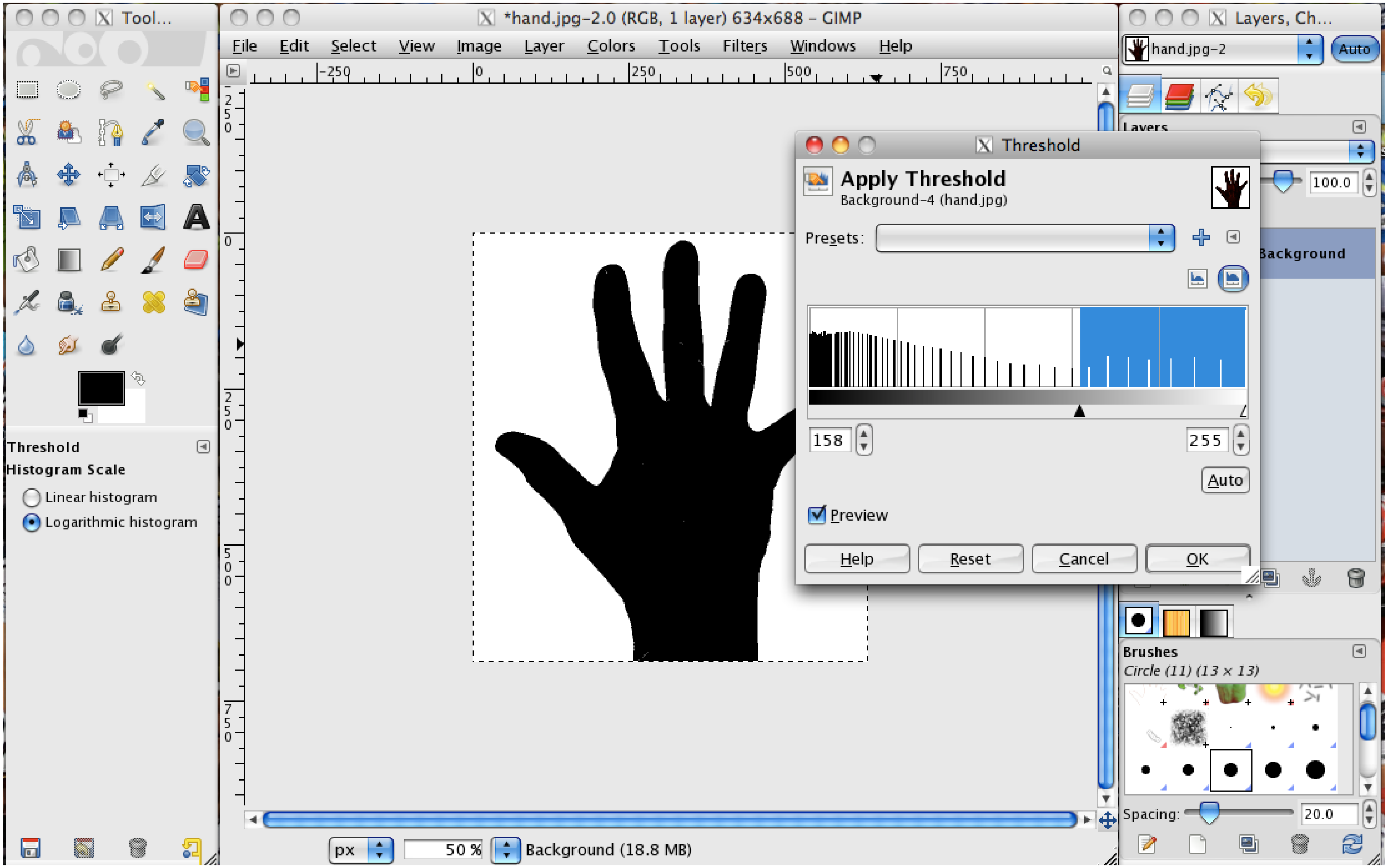

Supplementary Figure 7. STEP 3b: Move the histogram sliders so that the 2nd slider (grey) is in the notch between histogram nodes. If no notch, slide to the far right.

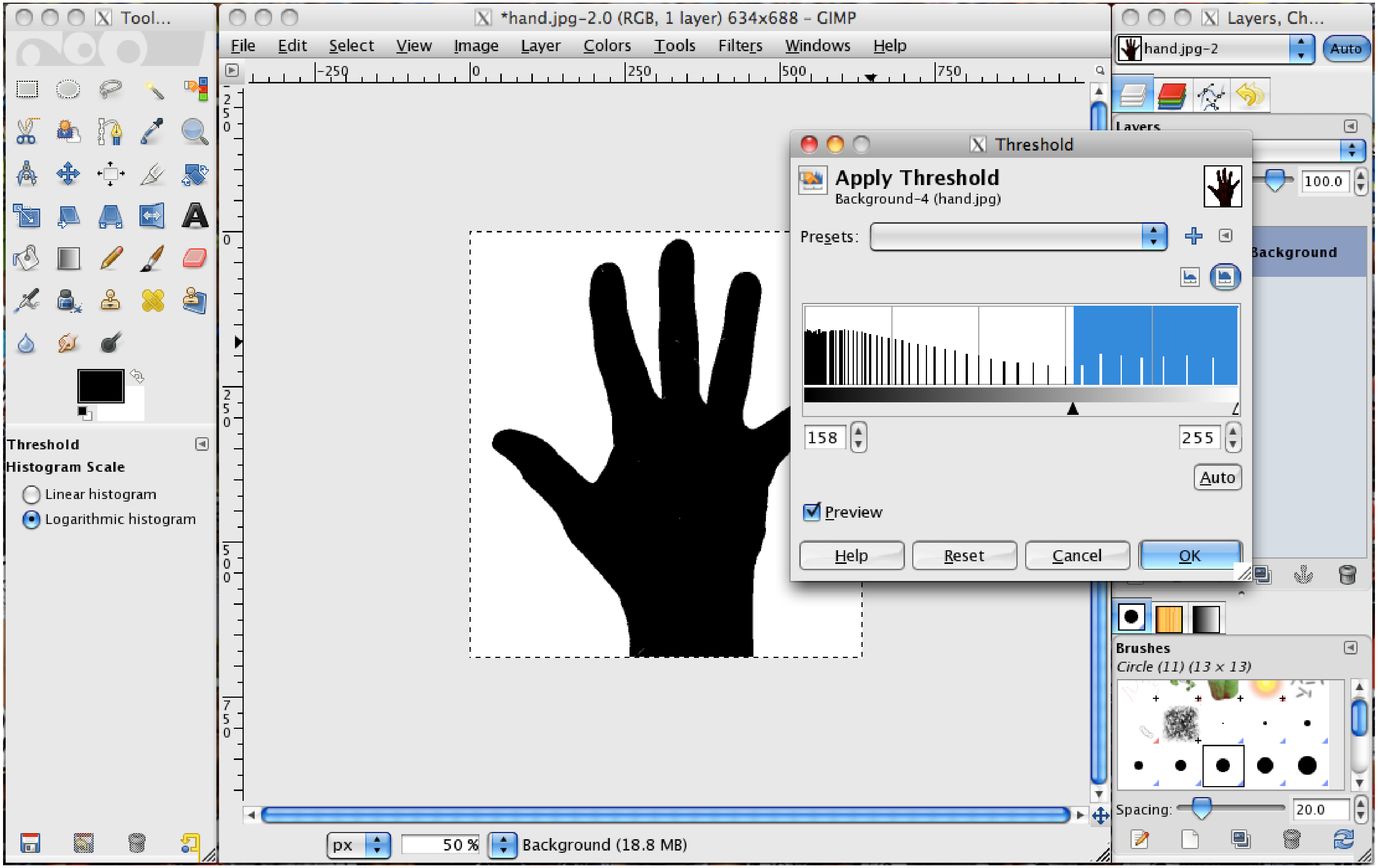

Supplementary Figure 8. STEP 3c: Click 'OK'.

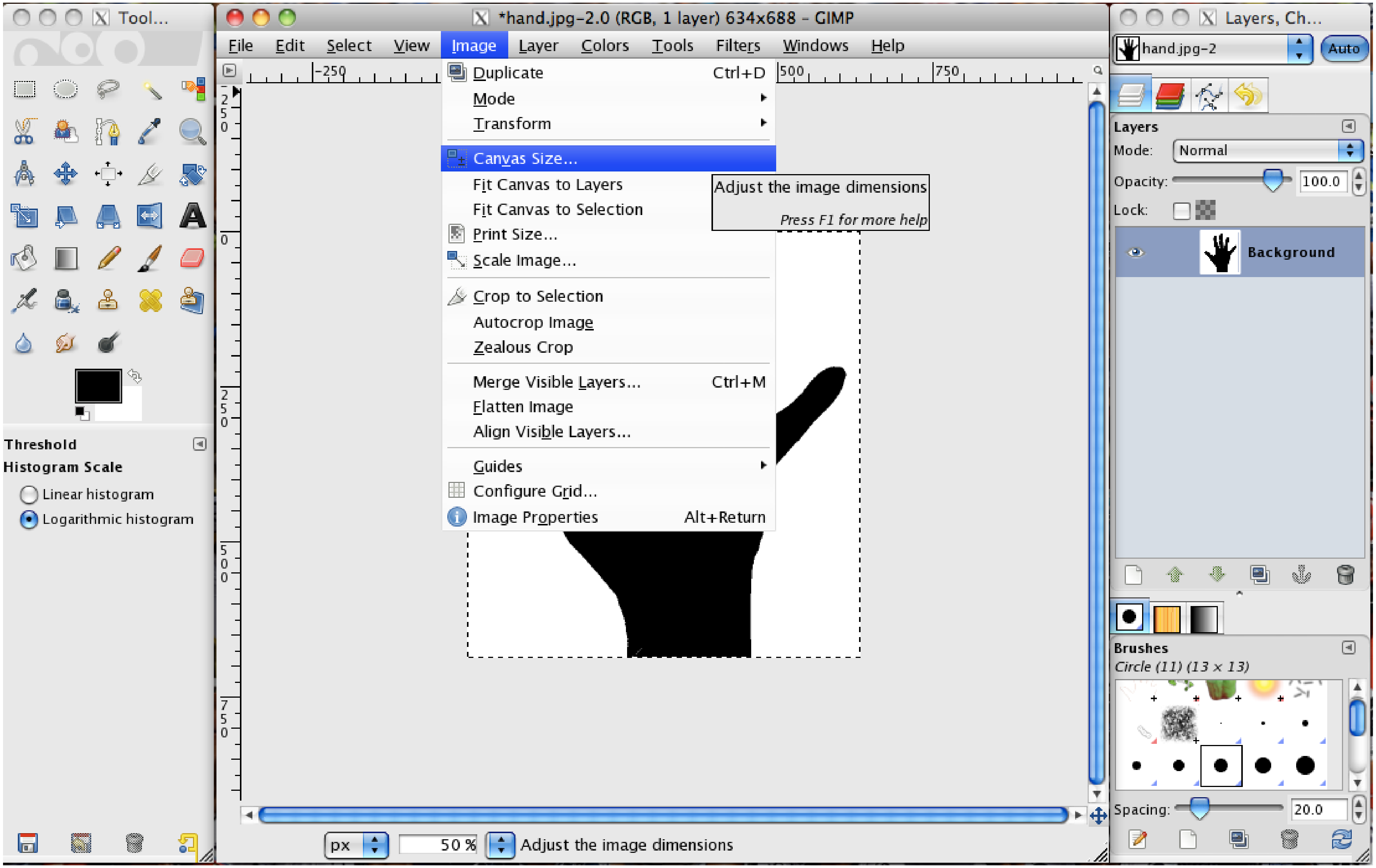

Supplementary Figure 9. STEP 4a: Access the canvas size tool from Image > Canvas Size menu.

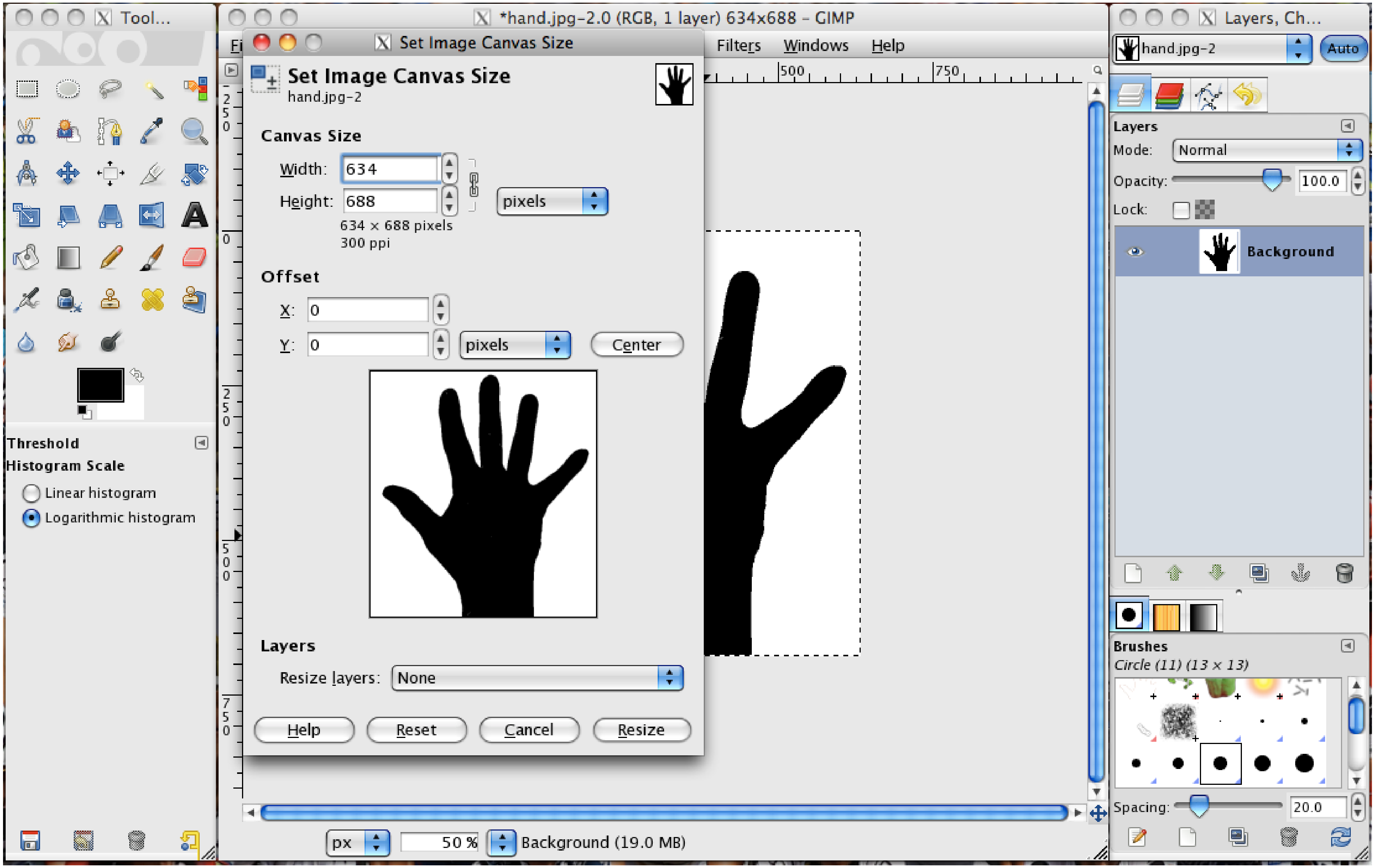

Supplementary Figure 20. STEP 4b: The Canvas Size tool.

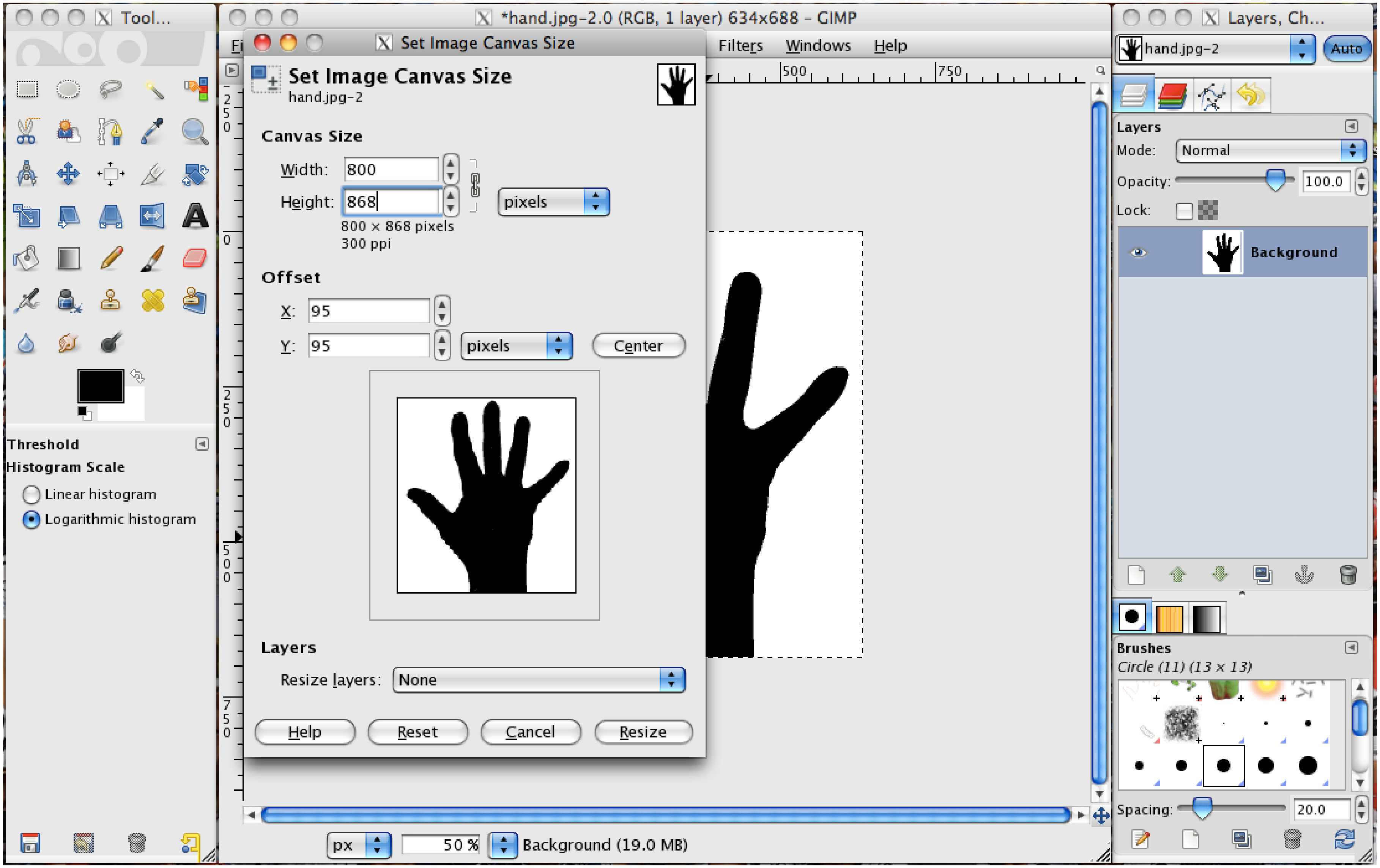

Supplementary Figure 31. STEP 4c: Increase the canvas size to an arbitrarily large size; utilize the offset to place the current image in the center of the canvas.

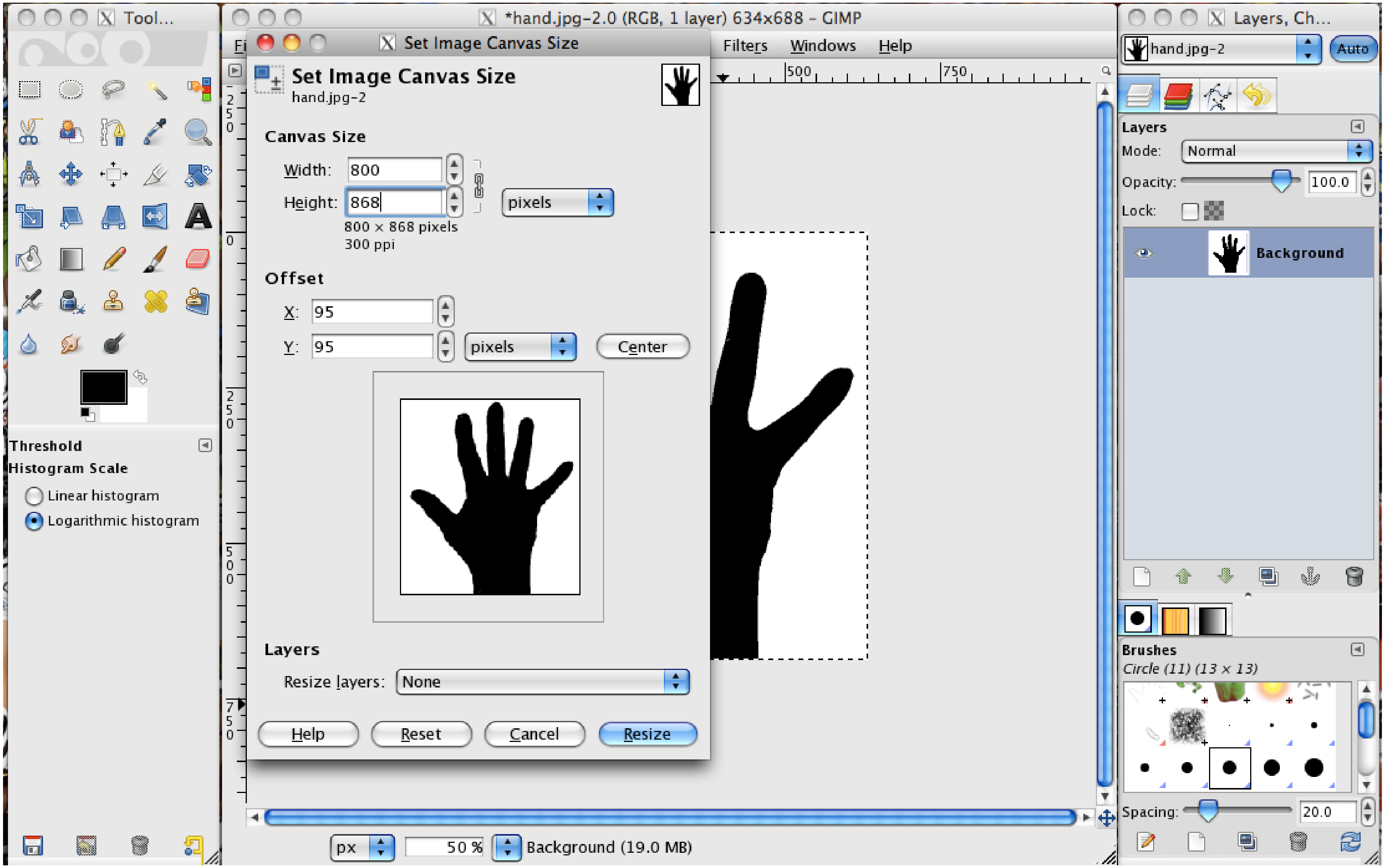

Supplementary Figure 42. STEP 4d: Click 'OK'.

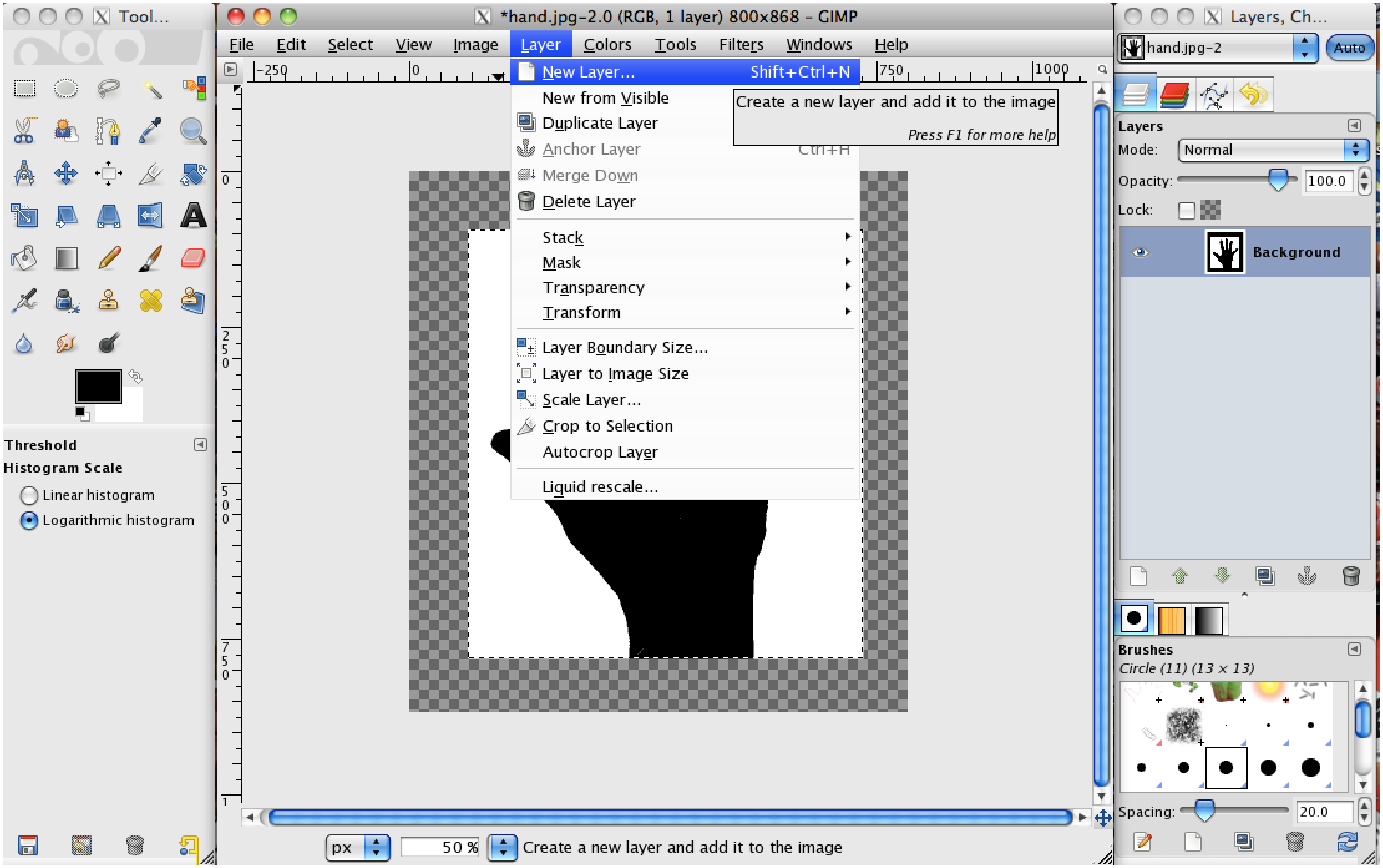

Supplementary Figure 53. STEP 4e: Access the New Layer tool via Layer > New Layer.

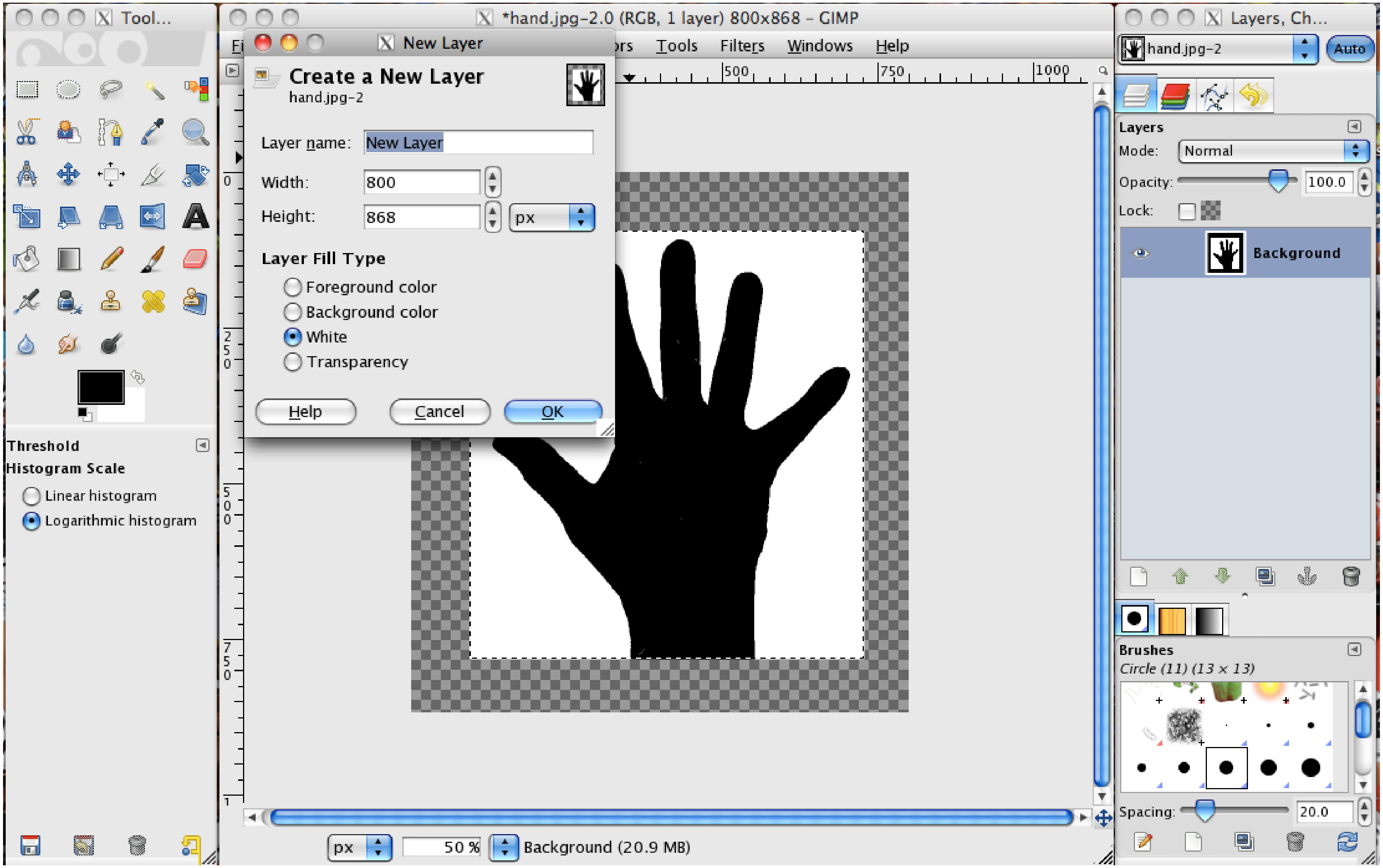

Supplementary Figure 64. STEP 4f: Create a new White Layer; Click 'OK'.

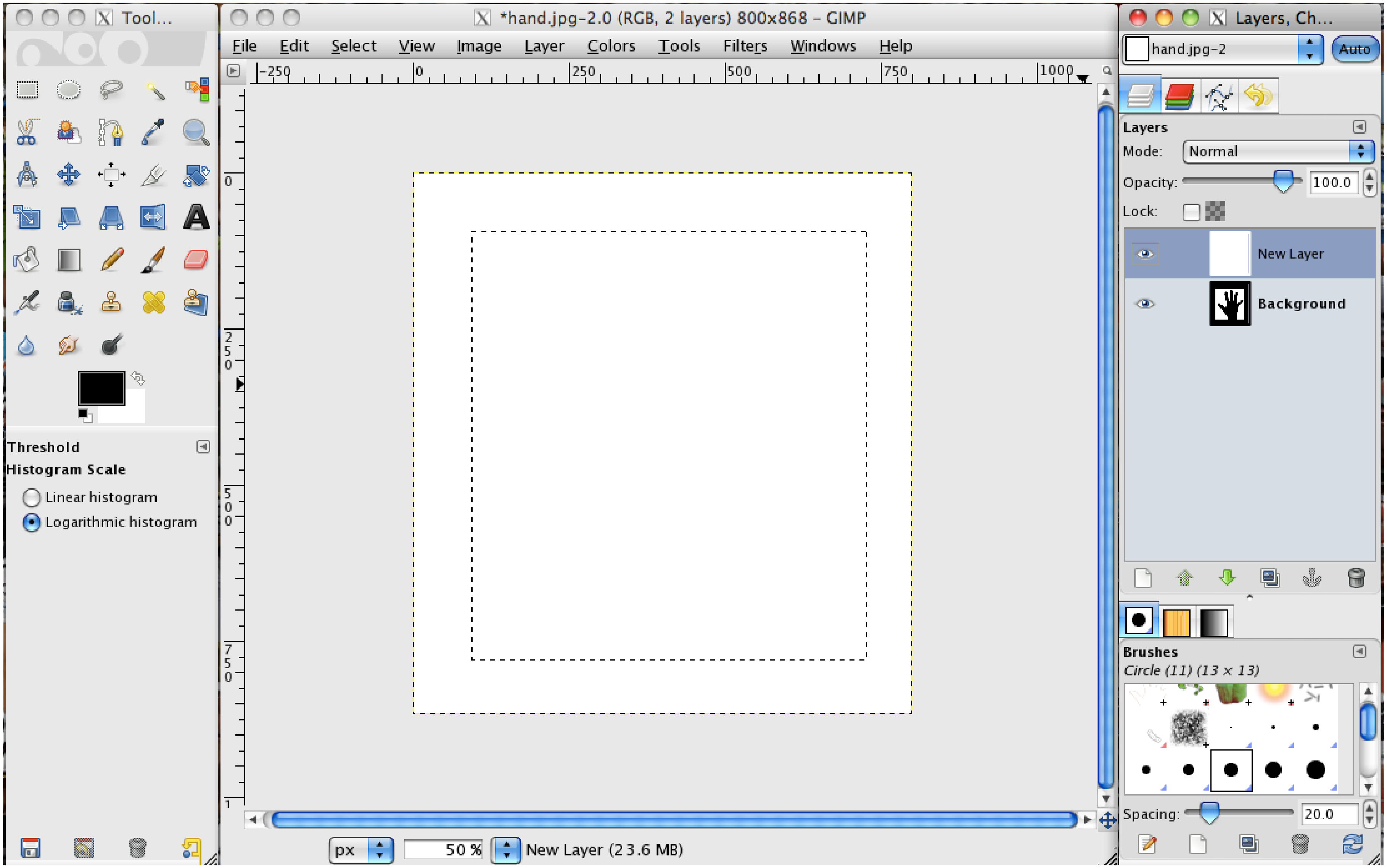

Supplementary Figure 75. STEP 4f: Layer will appear by default on top of the existing hand layer (notice Layers menu at right).

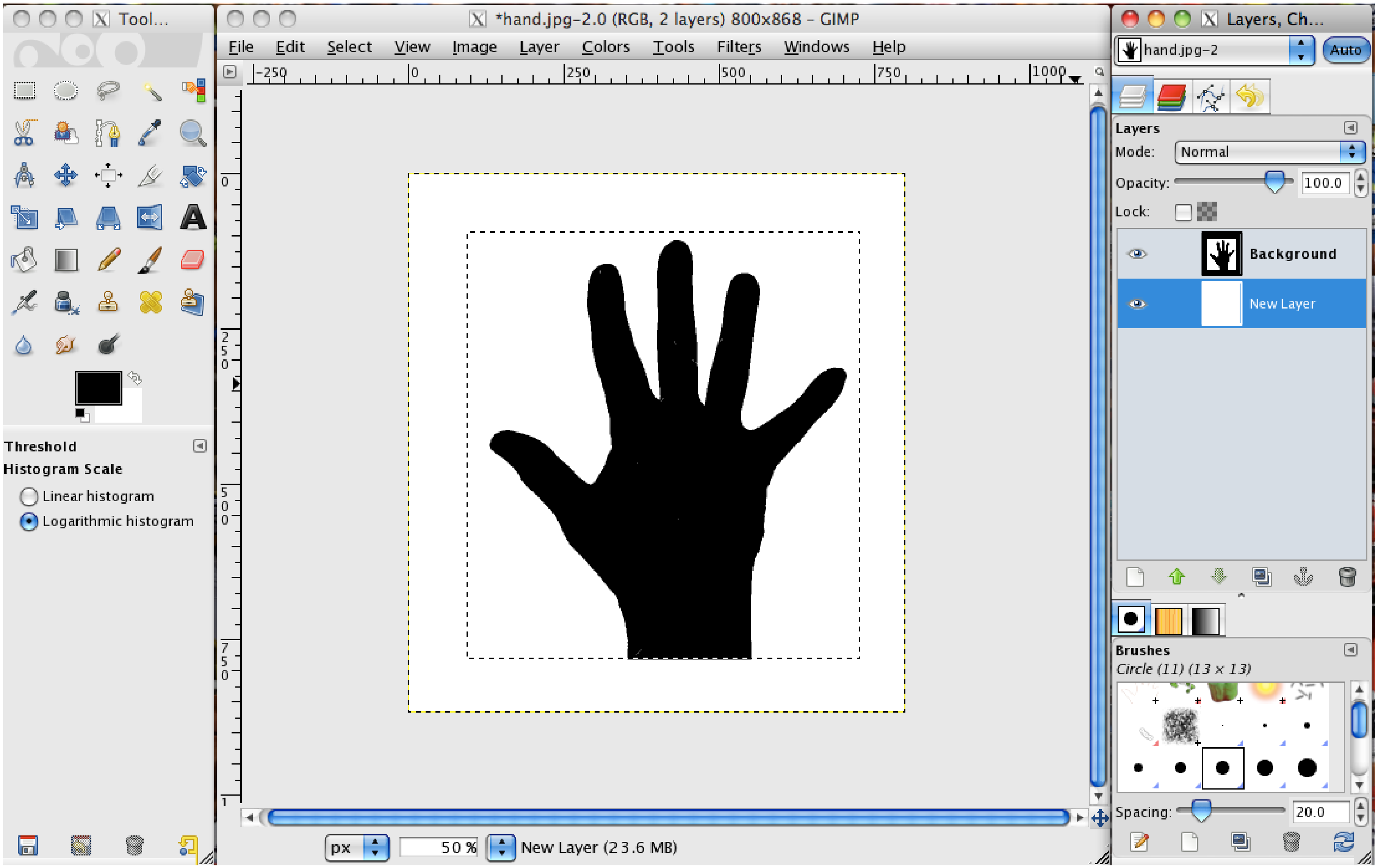

Supplementary Figure 86. STEP 4g: Move new white layer underneath the hand layer.

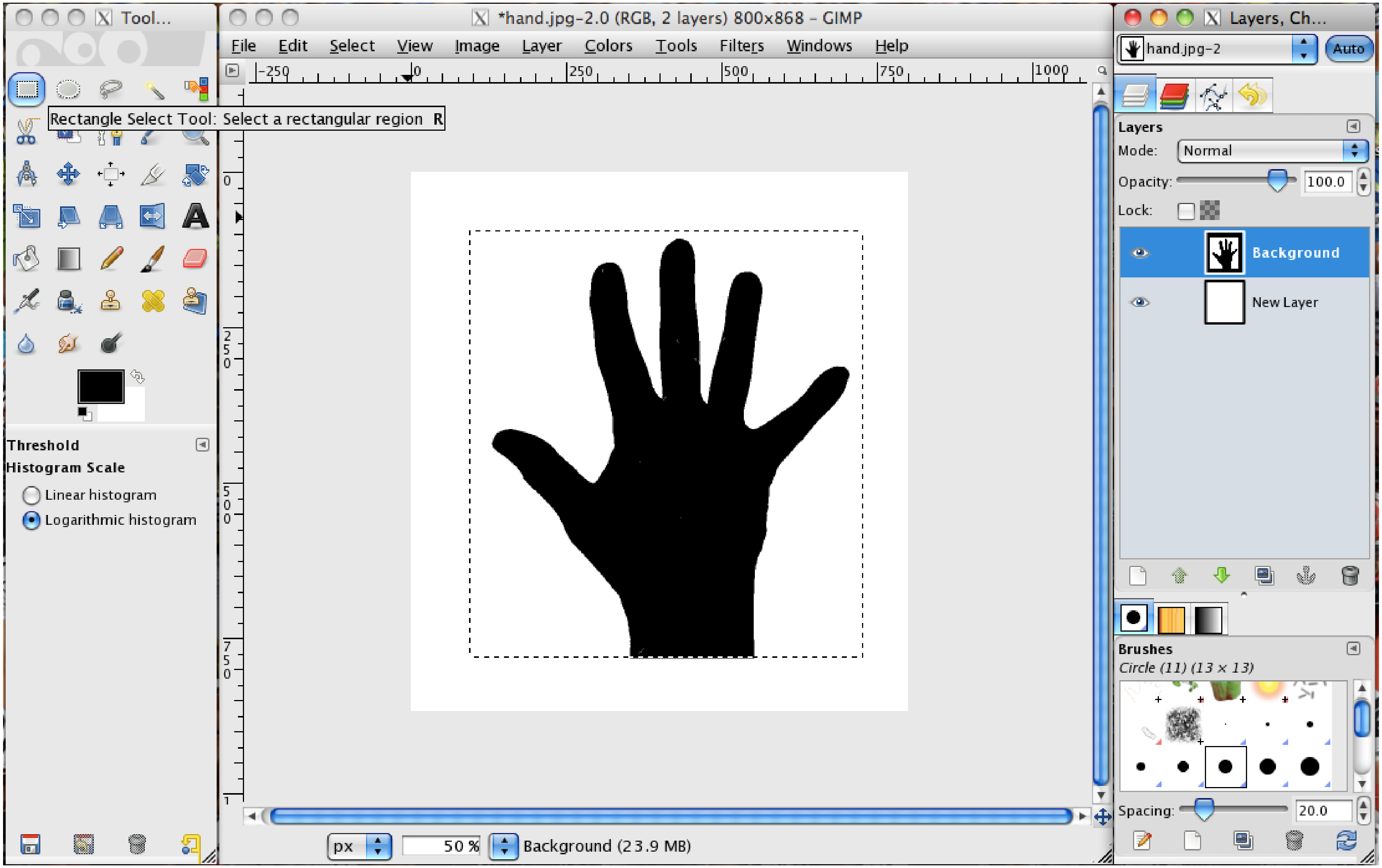

Supplementary Figure 97. STEP 4h: Access the Rectangle Select Tool from the Tool menu at left.

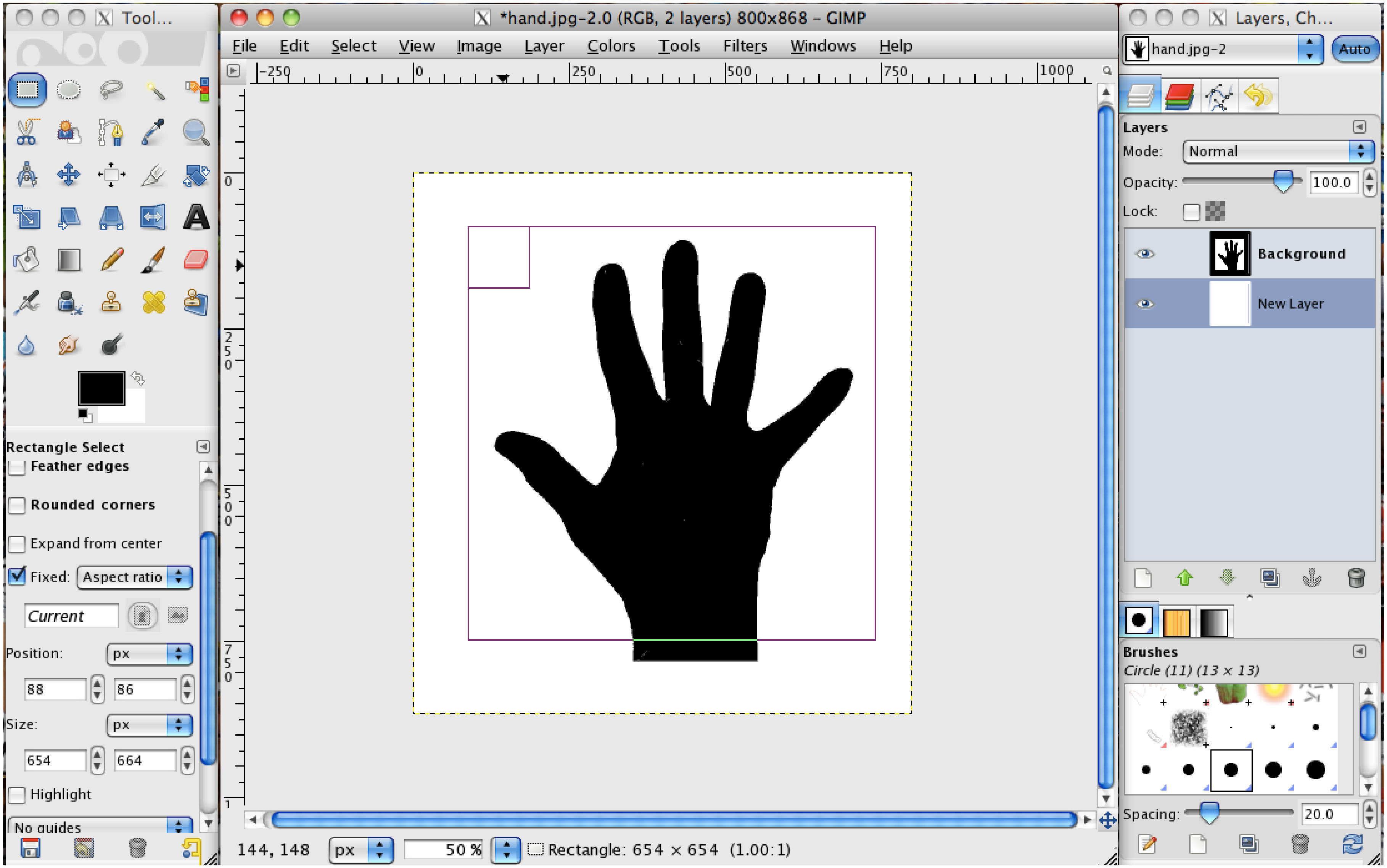

Supplementary Figure 108. STEP 4i: Drag a square section: Size is visible at the bottom of the Tool menu (shows 654 x 664 pixels here; not quite square....)

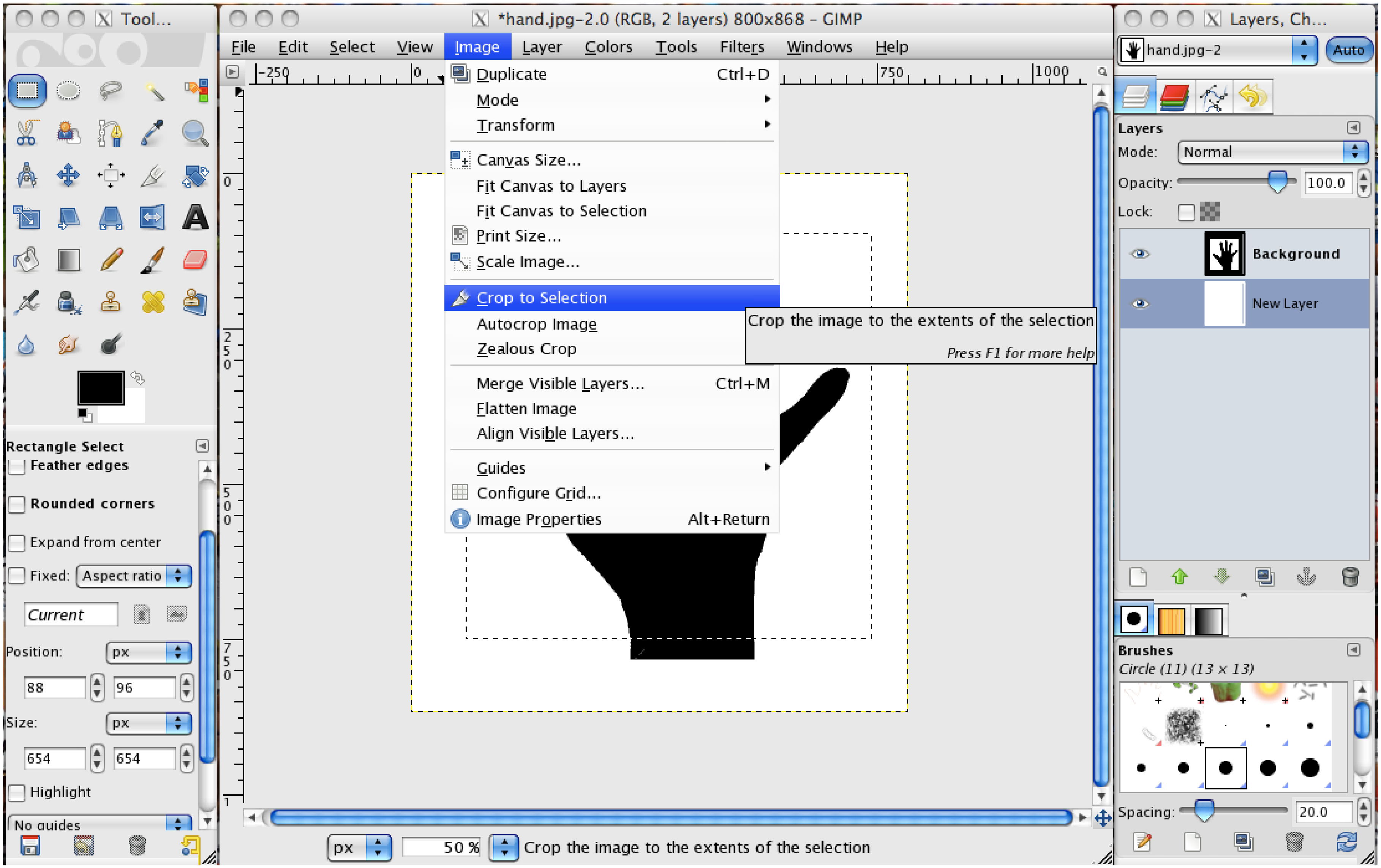

Supplementary Figure 119. STEP 4j: Crop to selection via Image > Crop to Selection (notice the Tool menu shows a square selection now: 654 x 654 pixels).

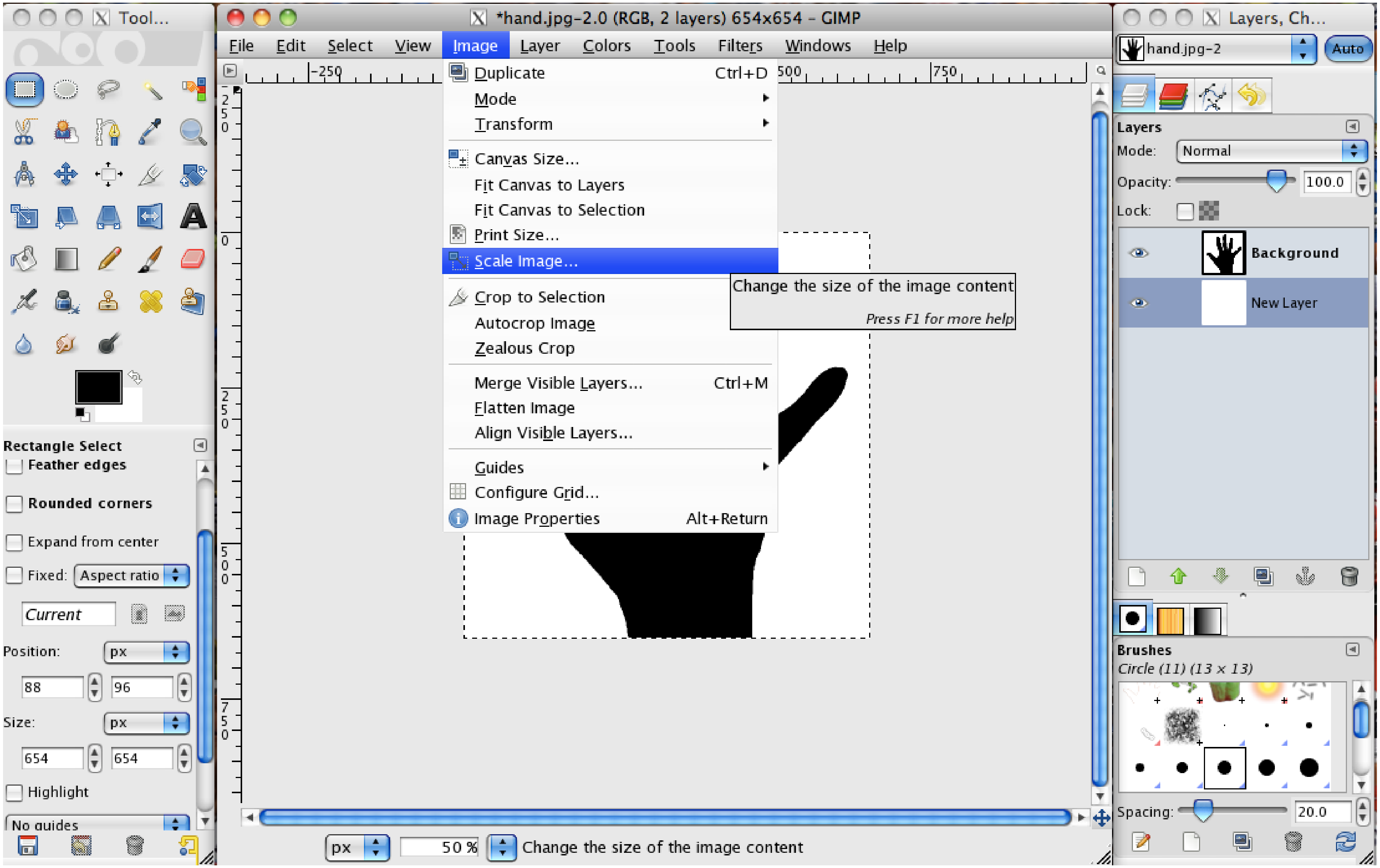

Supplementary Figure 20. STEP 4k: Access the image scaling tool from Image > Scale Image.

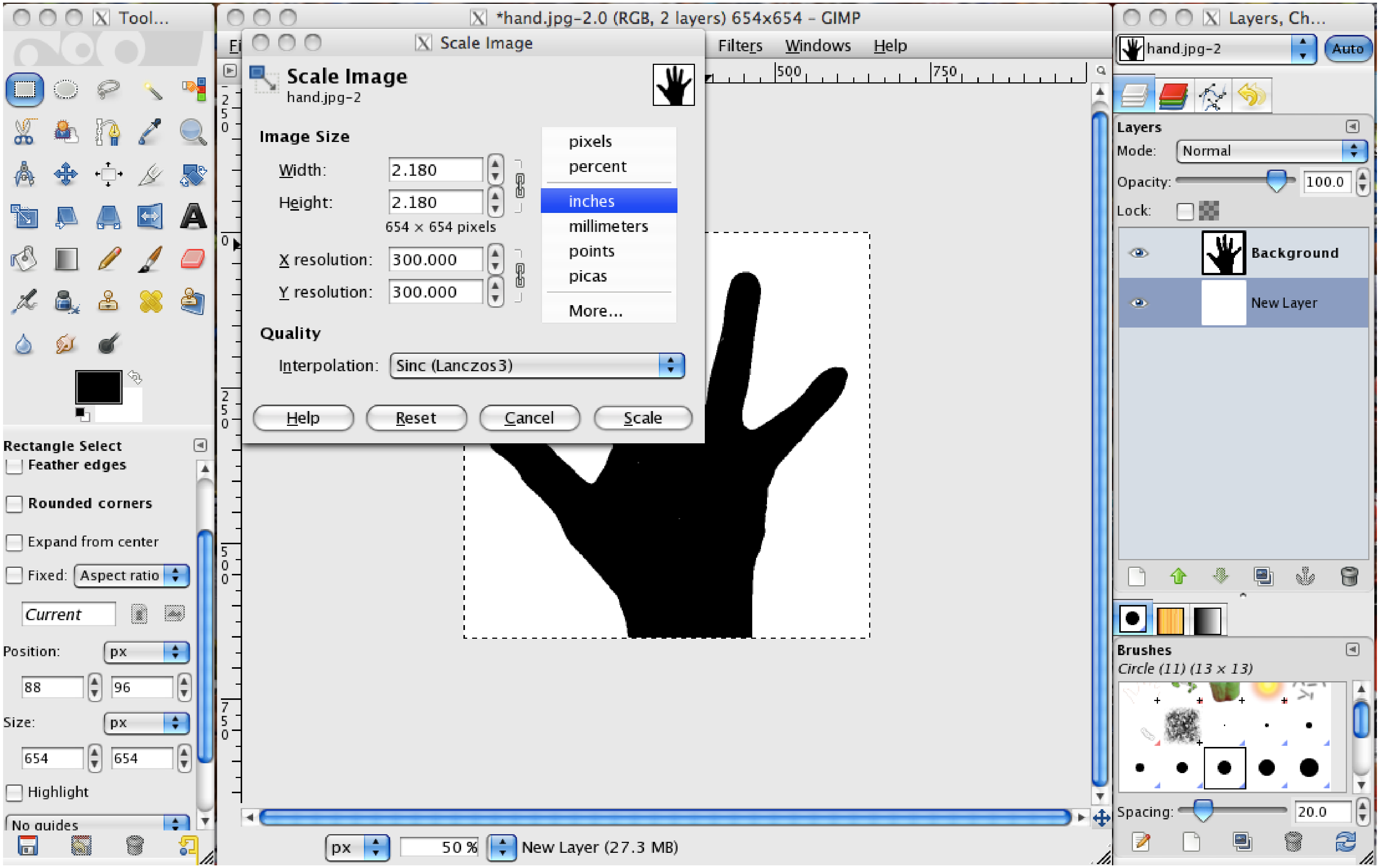

Supplementary Figure 21. STEP 4l: Convert units to pre-determined unit (inches selected here).

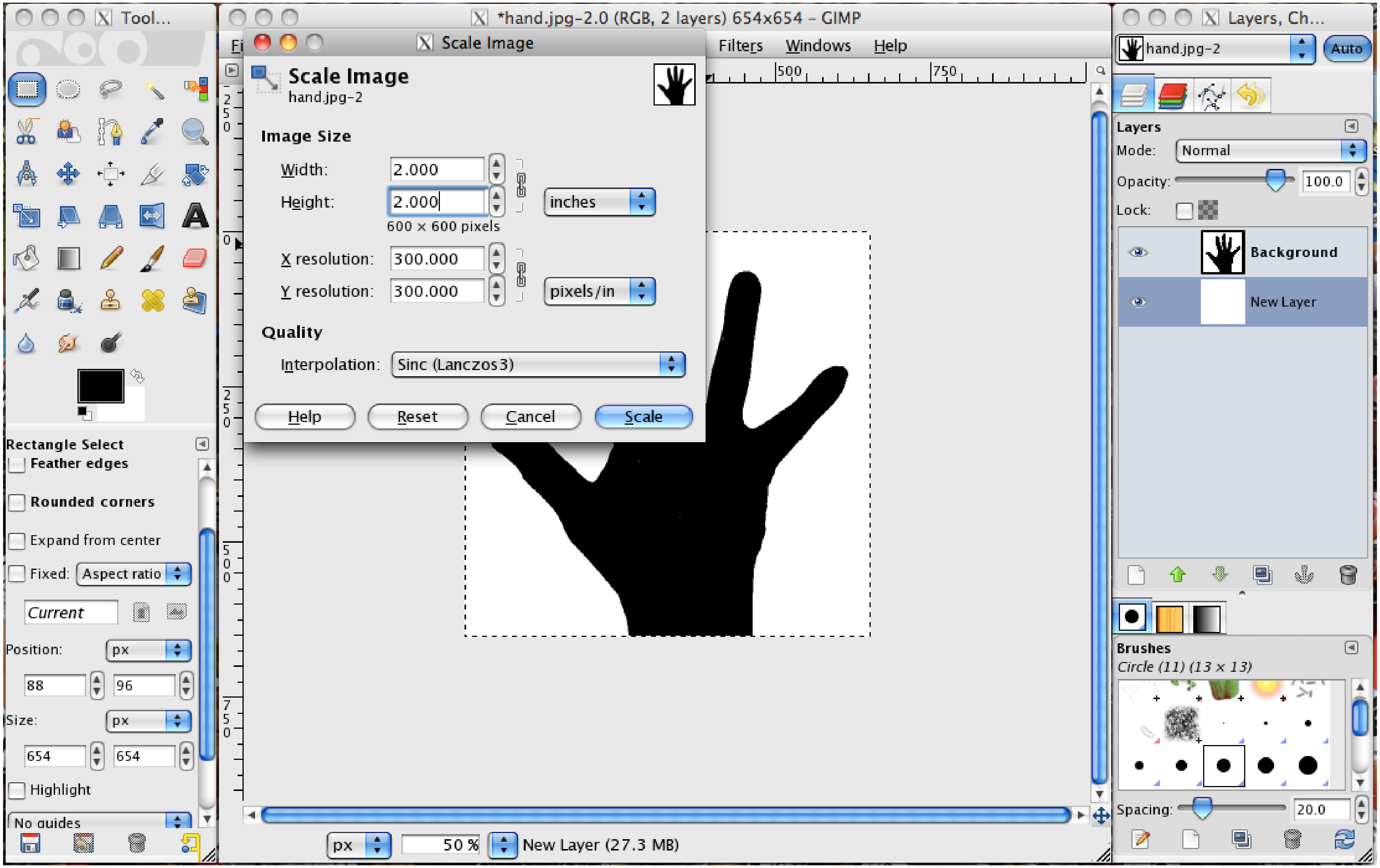

Supplementary Figure 22. STEP 4m: Specify size for this image to pre-determined value, here: 2 inches square. Click 'OK'.

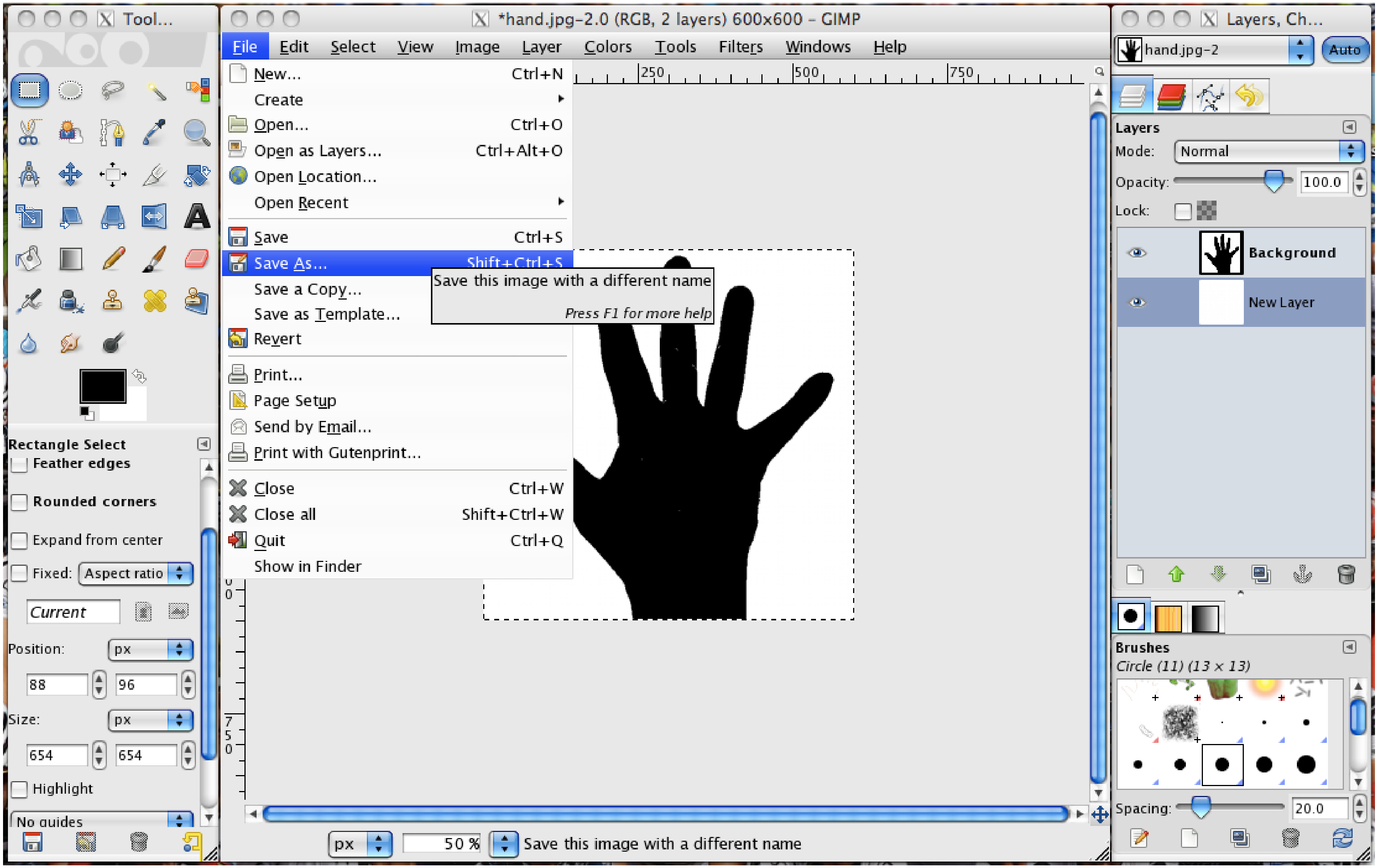

Supplementary Figure 23. STEP 5a: Access the save tool from File > Save As.

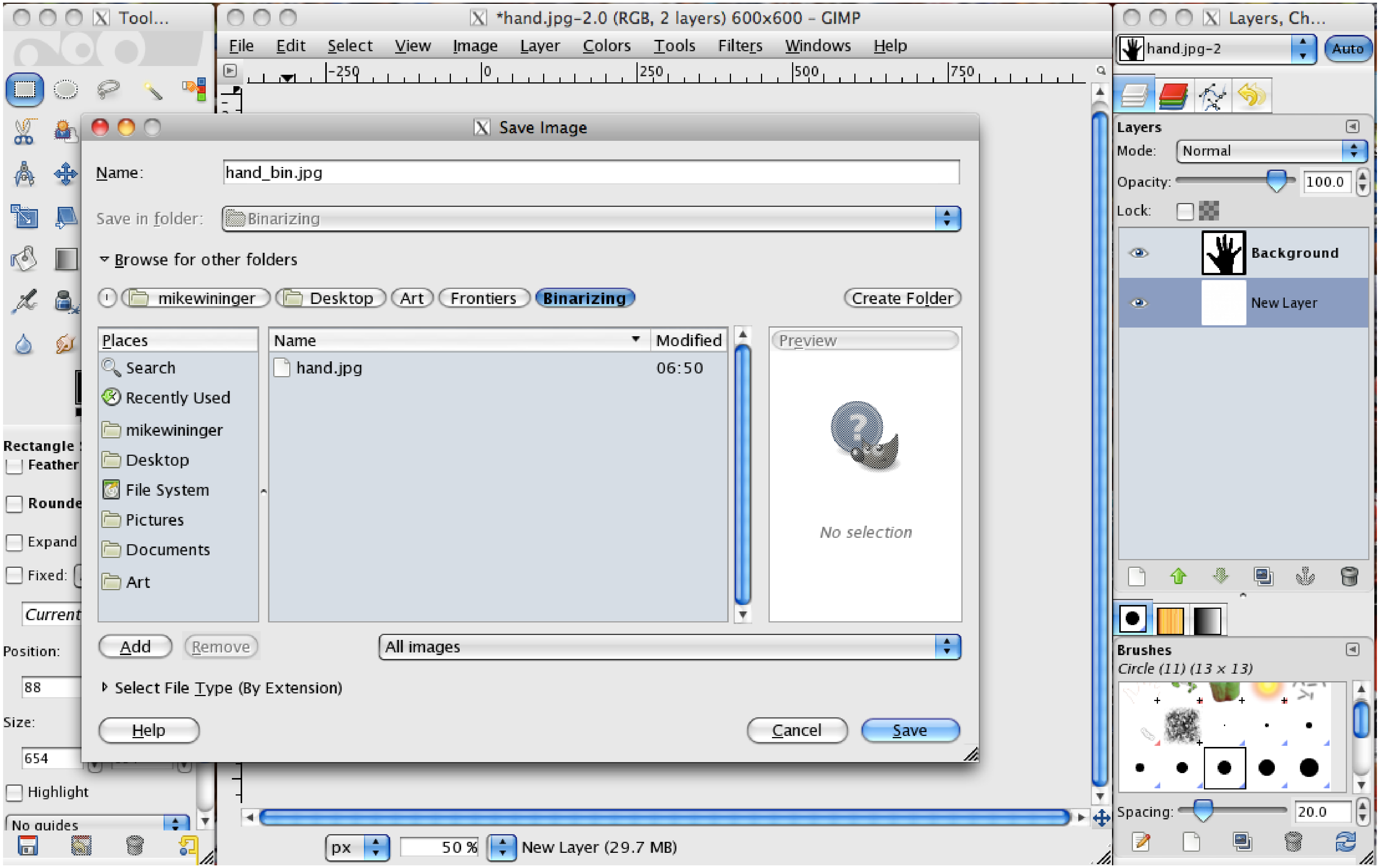

Supplementary Figure 24. STEP 5b: Save image under a different name (here suffixed with _bin).

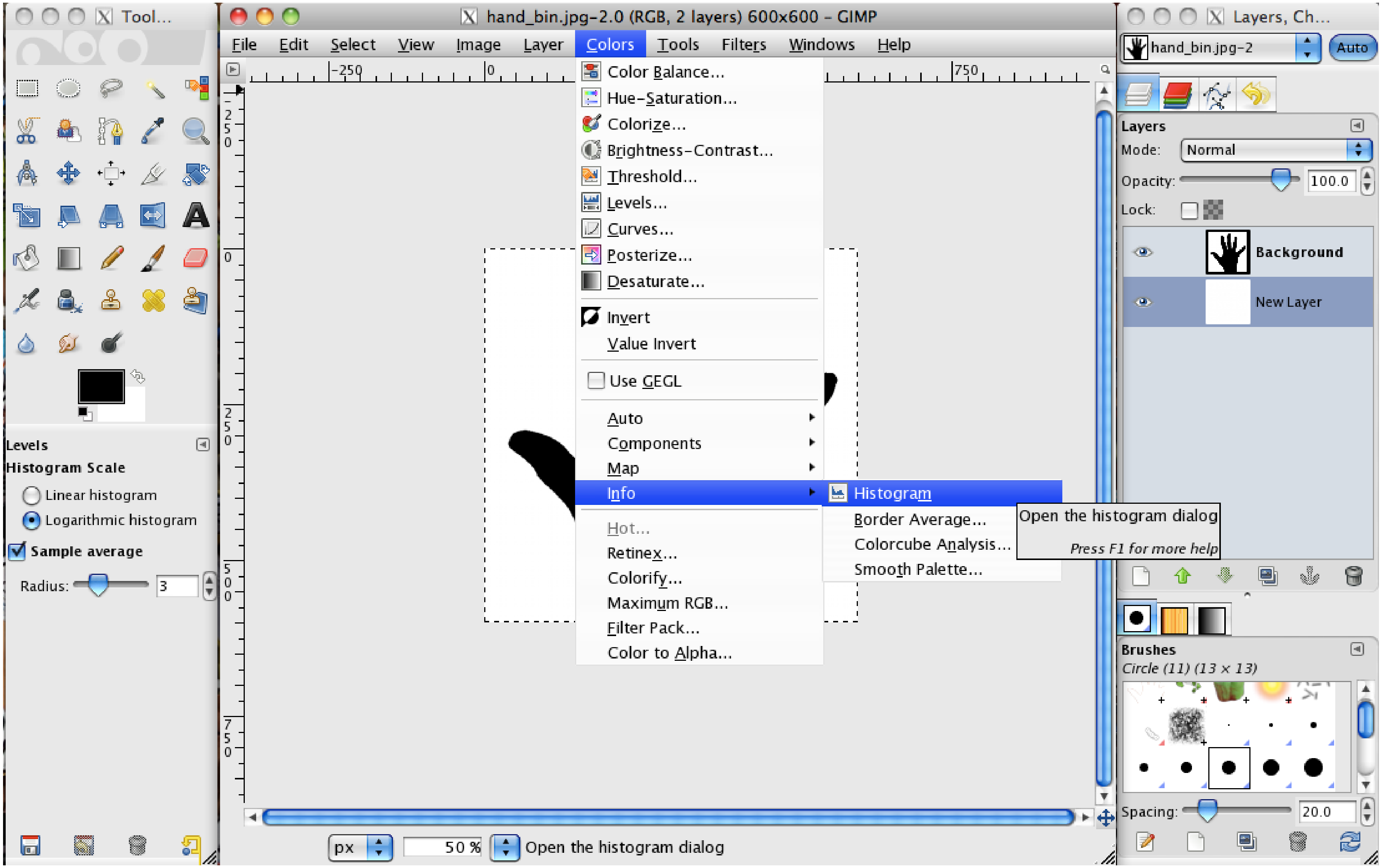

Supplementary Figure 25. STEP 6a: Access the pixel histogram tool from Colors > Info > Histogram.

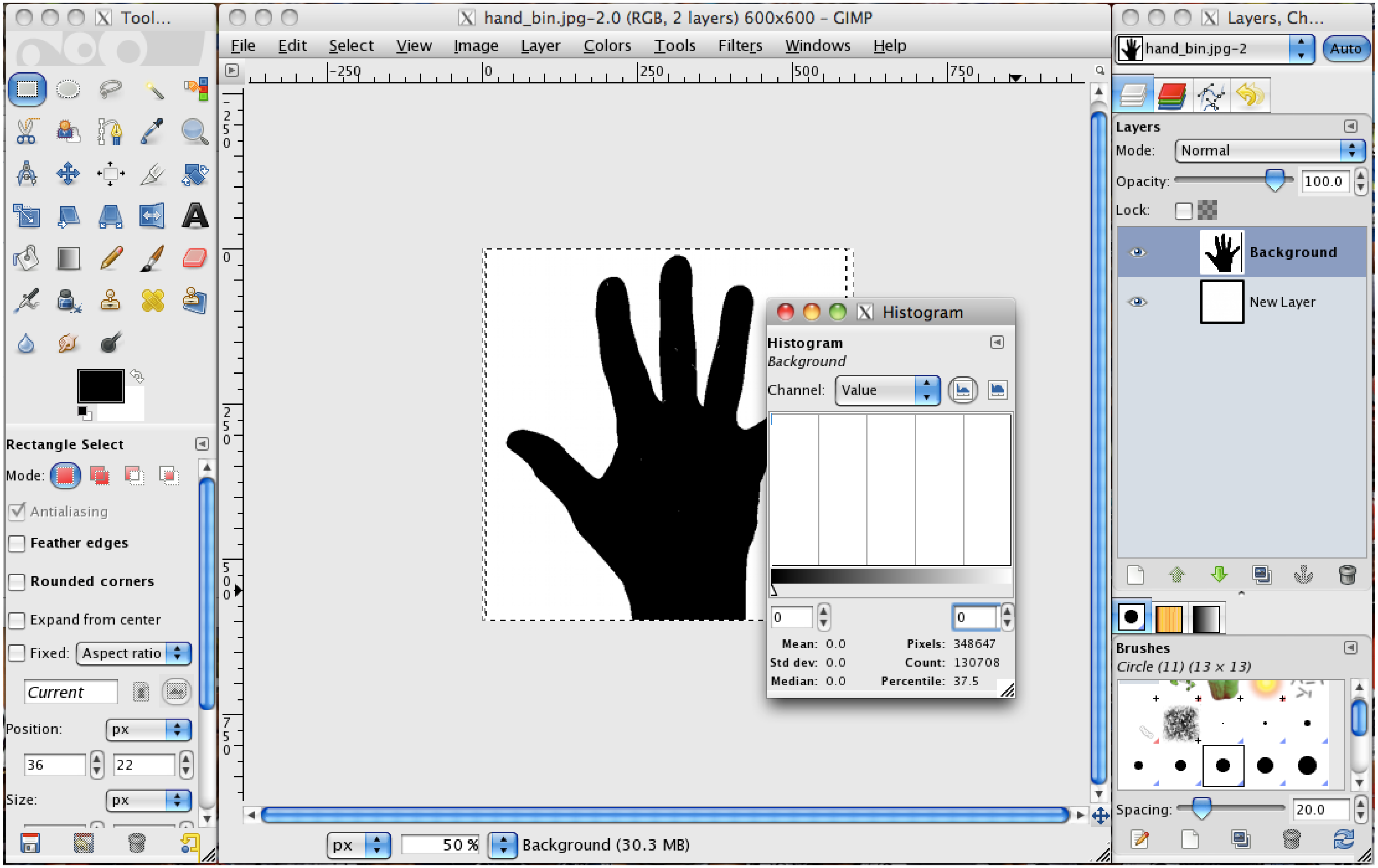

Supplementary Figure 26. STEP 5b: Specify the histogram range as counting only pixels 0 to 0 (two text boxes near bottom of tool). Note the pixel count (here: 130708 black pixels).
